# Effect of Expansion Media on Functional Characteristics of Bone Marrow-Derived Mesenchymal Stromal Cells

**DOI:** 10.3390/cells12162105

**Published:** 2023-08-19

**Authors:** Viktoria Jakl, Tanja Popp, Julian Haupt, Matthias Port, Reinhild Roesler, Sebastian Wiese, Benedikt Friemert, Markus T. Rojewski, Hubert Schrezenmeier

**Affiliations:** 1 Institute for Transfusion Medicine, University Hospital Ulm, 89081 Ulm, Germany; viktoria.jakl@uni-ulm.de (V.J.);; 2 Bundeswehr Institute of Radiobiology, 80937 Munich, Germanyjulian1haupt@bundeswehr.org (J.H.); matthiasport@bundeswehr.org (M.P.); 3 Clinic for Trauma Surgery and Orthopedics, Army Hospital Ulm, 89081 Ulm, Germany; 4 Core Unit of Mass Spectrometry and Proteomics, Ulm University Medical Center, 89081 Ulm, Germany; reinhild.roesler@uni-ulm.de (R.R.); sebastian.wiese@uni-ulm.de (S.W.); 5 Institute for Clinical Transfusion Medicine and Immunogenetics Ulm, German Red Cross Blood Donation Service Baden-Württemberg—Hessia and University Hospital Ulm, 89081 Ulm, Germany

**Keywords:** mesenchymal stromal cells, mesenchymal stem cells, media, serum-free, xenogeneic-free, platelet lysate

## Abstract

The therapeutic efficacy of mesenchymal stromal cells (MSCs) has been shown to rely on their immunomodulatory and regenerative properties. In order to obtain sufficient numbers of cells for clinical applications, MSCs have to be expanded ex vivo. Expansion media with xenogeneic-free (XF) growth-promoting supplements like human platelet lysate (PL) or serum- and xenogeneic-free (SF/XF) formulations have been established as safe and efficient, and both groups provide different beneficial qualities. In this study, MSCs were expanded in XF or SF/XF media as well as in mixtures thereof. MSCs cultured in these media were analyzed for phenotypic and functional properties. MSC expansion was optimal with SF/XF conditions when PL was present. Metabolic patterns, consumption of growth factors, and secretome of MSCs differed depending on the type and concentration of supplement. The lactate per glucose yield increased along with a higher proportion of PL. Many factors in the supernatant of cultured MSCs showed distinct patterns depending on the supplement (e.g., FGF-2, TGFβ, and insulin only in PL-expanded MSC, and leptin, sCD40L PDGF-AA only in SF/XF-expanded MSC). This also resulted in changes in cell characteristics like migratory potential. These findings support current approaches where growth media may be utilized for priming MSCs for specific therapeutic applications.

## 1. Introduction

Mesenchymal stromal cells (MSC) were first discovered by Friedenstein et al. in 1976 [1] and, since then, the interest in their medical use has increased continuously. MSCs can be isolated from various human tissues, including bone marrow (BM), adipose tissue, umbilical cord, and dental pulp, though general cell numbers are very low (e.g., about 0.001–0.01% of BM cells [2]). In 2006, the International Society for Cellular Therapy (ISCT) defined the minimal criteria for the heterogeneous cell population of MSCs. According to these criteria, MSCs have to express the surface antigen’s cluster of differentiation (CD)73, CD90, and CD105, and need to lack expression of common leucocyte and hematopoietic cell markers (e.g., CD45, CD34, CD14 or CD11b, CD79α, CD19, and major histocompatibility complex (MHC) II). Furthermore, MSCs have to adhere to plastic under standard culture conditions and need to show in vitro differentiation potential into cells of adipogenic, chondrogenic, and osteogenic lineages [3].

MSCs possess immunomodulatory and regenerative properties, thereby representing promising candidates for therapeutic use in a variety of diseases. Prominent areas of applications range from bone regeneration [4,5,6,7,8] or wound healing [9,10,11,12,13] to neurological disorders [14,15,16,17,18] and diseases based on disturbed immune responses like graft-versus-host disease (GvHD) [19,20,21,22,23,24,25]. Mechanisms of the therapeutic mode of action of MSCs are still not fully understood. However, due to studies that show trapping of MSCs in the lung as well as systemic clearance, hypotheses went from direct cell–cell contact-mediated mechanisms and engraftment of MSCs towards paracrine effects of MSC-derived factors like cytokines, chemokines, growth factors, or extracellular vesicles [26,27,28,29], also summarized as secretome [30].

In order to obtain sufficient cell numbers for clinical use, MSCs need to be expanded ex vivo. Sera like fetal bovine serum (FBS) can be used as growth-promoting cell culture supplements; however, animal-derived (xenogeneic) components are not desired for clinical applications due to the risk of disease transmission [31], immunization [32] and also reproducibility issues and ethical concerns [33]. Hence, human-derived xenogeneic-free (XF) substitutes like platelet lysate (PL) were developed. PL is manufactured from platelet concentrates and is mainly composed of albumin, immunoglobulins, and fibrinogen, in addition to various bioactive molecules including growth factors (e.g., insulin-like growth factor (IGF)-I, fibroblast growth factor (FGF)-2, platelet-derived growth factor (PDGF)-AA, PDGF-AB/BB, transforming growth factor (TGF)β), cytokines, and chemokines [34,35,36,37]. Although PL has been proven to be a safe and efficient cell culture supplement in many clinical applications [5,7,8,38], batch-to-batch variation may occur due to the pooling of platelet concentrates of different donors, which may affect MSC characteristics [39,40]. Hence, the request for defined serum- and xenogeneic-free (SF/XF) culture media have become more and more popular in recent years in order to have standardized culture conditions [41,42]. Media without serum often include growth factors also present in PL (e.g., FGF-2, PDGF, TGFβ) [43,44] or may still contain components derived from serum [45], though with consistency between different media batches. Several SF/XF media have been developed for MSC expansion, but exact media formulations are under lock and key in most cases due to their commercialization, and may vary between manufacturers. Accordingly, the divergent impact on MSC characteristics of different SF/XF media has been shown, and some of them even failed in efficiently supporting cell growth [46,47,48]. Varying conditions during cell cultures can also strongly affect MSC secretome in the conditioned media, and thus could be used as priming approaches, depending on therapeutic applications [49,50,51,52].

During this study, MSCs were expanded in media consisting of different ratios of αMEM supplemented with XF human PL (XF/PL) and an SF/XF medium. Using this approach, potential supportive properties of both media were combined and the effects of varying culture conditions on MSC proliferation and characteristics were investigated. Analyses revealed alterations in the basic metabolism of cells as shown by differences in the expression of proteins involved in various metabolic pathways, as well as divergent consumption of several growth factors. Changes in metabolic fundamentals may affect MSC functionality as indicated by altered secretion of functionally relevant factors and migratory potential of cells, and thus may be utilized for priming of MSCs by expansion media.

## 2. Materials and Methods

### 2.1. Cell Culture of MSCs

#### 2.1.1. Harvesting of Primary Material

Primary MSCs derived from BM were used for the experiments. Small-volume BM aspirates (approximately 25 mL to 35 mL from the iliac crest) were obtained from healthy volunteer donors according to standard operating procedures. Informed consent was obtained from all donors and the collection of the material was approved by the Ethical Committee of the University of Ulm (Ulm, Germany). MSCs were isolated by seeding BM into cell culture vessels as previously described by Rojewski et al. [38]. MSCs of the same donors were used for all media in order to exclude the impact of donor variability rather than the effects of different media.

#### 2.1.2. Cell Expansion in Different Media

The expansion media αMEM supplemented with 8% PL (IKT Ulm, Ulm, Germany) and 1 i.U. per mL heparin (Ratiopharm GmbH, Ulm, Germany) (αMEM+8%PL; medium 1), StemMACS^TM^ MSC Expansion Media Kit XF (Miltenyi Biotec B.V. & Co. KG, Bergisch Gladbach, Germany) (StemMACS^TM^; medium 13), or mixtures of different ratios of both media (media 2–12; Table 1) were used for the experiments.

In the first step, expansion of cells was compared for media 1 to 13. For these experiments, MSCs primarily isolated in medium 1 were used for all other media approaches. Briefly, MSCs of passage 0 (P0) were thawed and expanded for passage 1 (P1) in T500 triple flasks in medium 1 in order to reduce stress after thawing. Then, expansion of passage 2 (P2) and passage 3 (P3) was performed in media 1 to 13 in T175 flasks using a seeding density of 2.000 cells/cm^2^. Only MSCs of P3 were used for analyses that included determination of expansion parameters, viability of cells, and basic flow cytometric characterization. In addition, these cells were used for scratch wound migration assay.

In the second step, MSCs were isolated from BM in media 1, 4, 7, 10, and 13, as these media seemed to be most interesting during the first step. These MSCs were seeded at 2.000 cells/cm^2^ in T175 flasks and expanded for P1 in respective isolation media. Expansion parameters and viability of cells were determined and basic flow cytometric characterization was performed. These cells were used for all further experiments except for scratch wound migration assay.

Harvesting of cells was performed on the same day for all media by using TrypZean^TM^ (Lonza Group Ltd., Basel, Switzerland) for detachment of cells. Cell count was determined by using a Neubauer chamber (Glaswarenfabrik Karl Hecht GmbH & Co. KG, Sondheim vor der Rhön, Germany), and identification of dead cells was achieved by trypan blue staining (Sigma-Aldrich Chemie GmbH, Taufkirchen, Germany). The expansion parameters harvesting density, doubling time, and number of population doublings were calculated. Viability of cells was determined by the ratio of living cells to total cells (including living and dead cells). During the cell cultures, samples of conditioned media were taken at media exchange and harvesting of cells for further analyses. Samples of media were taken prior to the cell cultures. All samples were centrifuged for 5 min at 14.000× *g* and room temperature (RT) for removal of cellular debris or large particles and subsequently frozen at −80 °C.

#### 2.1.3. Determination of Glucose Consumption, Lactate Generation and Yield

Glucose and lactate concentrations were determined in media at the beginning of and accordingly during the expansion of the cells in conditioned media by a CONTOUR^®^XT (Ascensia Diabetes Care Deutschland GmbH, Leverkusen, Germany) and Lactate Plus Meter (Nova Biomedical Corporation, Waltham, MA, USA), respectively. Measured values below detection thresholds were assumed to be zero. Glucose consumption and lactate generation were calculated by the difference in respective concentrations in media and conditioned media. Both parameters were normalized to one million harvested cells and a time interval of one day. The yield of lactate from glucose was obtained by division of lactate generation by glucose consumption as described by Schop et al. [53].

### 2.2. Characterization of MSCs

#### 2.2.1. Flow Cytometric Characterization of MSCs

Surface antigen expression of MSCs was analyzed by flow cytometry. The following antibodies were used for the analyses: CD3 (clone SK7; BD Biosciences, Franklin Lakes, NJ, USA or BioLegend, San Diego, CA, USA), CD9 (clone M-L13; BD Biosciences), CD10 (clone HI10a; BD Biosciences), CD13 (clone WM15; BD Biosciences), CD14 (clone MØP9; BD Biosciences or clone M5E2 or HCD14; both from BioLegend), CD29 (clone TS2/16; Thermo Fisher Scientific Inc., Waltham, MA, USA), CD31 or platelet/endothelial cell adhesion molecule 1 (PECAM-1) (clone WM59; BD Biosciences or BioLegend), CD34 (clone 8G12; BD Biosciences), CD36 (clone AC106; Miltenyi Biotec B.V. & Co. KG), CD44 or homing cell adhesion molecule (HCAM) (clone G44-26; BD Biosciences), CD45 (clone 2D1 or HI30; both from BD Biosciences), CD49a (clone SR84; BD Biosciences), CD49c (clone C3 II.1; BD Biosciences), CD49d (clone 9F10; BD Biosciences), CD49e (clone IIA1; BD Biosciences), CD49f (clone GoH3; BD Biosciences), CD51 (clone NKI-M9; BioLegend), CD61 (clone VI-PL2; BD Biosciences), CD63 (clone H5C6; BD Biosciences), CD73 (clone AD2; BD Biosciences), CD81 (clone JS-81; BD Biosciences), CD90 (clone 5E10; BD Biosciences), CD105 (clone SN6; Bio-Rad AbD Serotec GmbH, Puchheim, Germany or clone 266; BD Biosciences), CD140a or platelet-derived growth factor receptor (PDGFR)A (clone 16A1; BioLegend), CD140b or PDGFRB (clone 28D4; BD Biosciences), CD146 or melanoma cell adhesion molecule (MCAM) (clone P1H12; BD Biosciences), CD220 or insulin receptor (INSR) (clone 3B6/IR; BD Biosciences), CD221 or IGF-I receptor (IGF1R) (clone 1H7; BioLegend), CD222 or IGF-II receptor (IGF2R) (clone QA19A18; BioLegend), CD271 or nerve growth factor receptor (NGFR) (clone ME20.4; BioLegend), CD331 or fibroblast growth factor receptor (FGFR)1 (clone M17A3; Novus Biologicals, LLC, Centennial, CO, USA), CD332 or FGFR2 (clone #98725; R&D Systems, Inc., Minneapolis, MN, USA), CD333 or FGFR3 (clone #136334; R&D Systems, Inc.), CD362 or Syndecan-2 (clone #305515R; R&D Systems, Inc.), epidermal growth factor receptor (EGFR) (clone AY13; BioLegend), glucose transporter (GLUT)1 (clone 202915; BD Biosciences), GLUT3 (clone #202017; R&D Systems, Inc.), GLUT4 (clone #925932; R&D Systems, Inc.), MHC I (clone G46-2.6; BD Biosciences), MHC II (clone Tu39; BD Biosciences), mesenchymal stromal cell antigen-1 (MSCA1) (clone W8B2; Miltenyi Biotec B.V. & Co. KG) and stimulated by retinoic acid 6 (STRA6) (clone #496613; R&D Systems, Inc.). Staining of cells was performed as per manufacturer’s instructions in a standard panel including identity markers (CD73, CD90, CD105) and purity markers (CD14, CD34, CD45, MHC II) and in an extended panel including all of the other surface antigens (for staining details, see Tables S1–S3). Mean fluorescence intensities were measured using a FACScan^TM^ system with BD CellQuest^TM^ software (version 3.3; BD Biosciences) or a FACSCelesta^TM^ Cell Analyzer with BD FACSDiva^TM^ software (version 8.0.1.1; BD Biosciences).

#### 2.2.2. Differentiation Assays

MSCs grown in media 1, 4, 7, 10, and 13 were differentiated into cells of adipogenic, chondrogenic, and osteogenic lineages. The differentiation assay kits Human Mesenchymal Stem Cell (hMSC) Adipogenic Differentiation Medium BulletKit^TM^ (Lonza Group Ltd.), StemMACS^TM^ ChondroDiff Media, Human and StemMACS^TM^ OsteoDiff Media, and Human (both from Miltenyi Biotec B.V. & Co. KG) were used as per manufacturer’s instructions. In brief, cells were thawed and seeded into SlideFlasks (Thermo Fisher Scientific Inc.) at densities of 200.000 cells/cm^2^ for adipogenic and 45.000 cells/cm^2^ for chondrogenic and osteogenic differentiation, respectively. Cells grown in αMEM with 20% FBS (Biological Industries, Kibbutz Beit-Haemek, Israeal) served as a control. The medium was exchanged every 2–3 days. When differentiation was completed, cells were stained by Oil Red O and hematoxylin for adipogenic differentiation (Sigma-Aldrich Chemie GmbH) and methylene blue for chondrogenic differentiation (Sigma-Aldrich Chemie GmbH). Activity of alkaline phosphatase was visualized by 5-bromo-4-chloro-3-indolylphosphate (BCIP)/nitroblue tetrazolium (NBT) substrate for osteogenic differentiation (Sigma-Aldrich Chemie GmbH). Pictures of stained cells were taken using an inverted phase contrast microscope (BZ-X710; KEYENCE DEUTSCHLAND GmbH, Neu-Isenburg, Germany) with BZ-X Viewer software (version 01.03.01.01).

#### 2.2.3. Scratch Wound Migration Assay

The migration potential of MSCs grown in media 1, 4, 7, 10, and 13 was investigated. Cells were thawed and expanded for one passage in T75 flasks in the respective media. Then, cells were seeded into IncuCyte^®^ ImageLock 96-well plates (Sartorius AG, Göttingen, Germany) at densities of 12.000 cells/cm^2^ in quadruplicates (n = 4). Cells were allowed to adhere for 24 h before the scratch wound area was created by the IncuCyte^®^ Wound Maker (Sartorius AG). Migration of cells into the wound area was monitored in the IncuCyte^®^ S3 Live-Cell Analysis system (Sartorius AG). Pictures were taken every 2 h for 4 d and analysis was performed by IncuCyte^®^ Software (version 2019B Rev2; Sartorius AG). Migration potential was evaluated by calculating the relative wound density. The software measured the spatial cell density in the wound area relative to the spatial cell density outside the wound area at every time point. Thus, stronger proliferation of cells in different media as a confounding factor for migration can be excluded.

### 2.3. Proteome Analysis of MSCs and Media

For label-free analysis, 20 μg of protein was reduced with 5 mM dithiothreitol (DTT) (AppliChem GmbH, Darmstadt, Germany) for 20 min at RT and subsequently alkylated with iodoacetamide (Sigma-Aldrich Chemie GmbH) for 20 min at 37 °C. Trypsin (Thermo Fisher Scientific Inc.) was added in a 1:50 enzyme–protein ratio and digested overnight at 37 °C. Employing an Orbitrap Elite (Thermo Fisher Scientific Inc.) mass spectrometer online coupled to an RSLCnano (Thermo Fisher Scientific Inc.), samples were analyzed as described previously [54].

For tandem mass tag (TMT) labeling, 100 µg of sample was labeled using TMT (Thermo Fisher Scientific Inc.) according to the manufacturer’s protocol. Following equal mixing, combined samples were fractionated using strong cation exchange (SCX) chromatography on a BioRSLC (Thermo Fisher Scientific Inc.). Fourteen fractions were collected and desalted on OASIS cartridges (Waters GmbH, Eschborn, Germany) according to protocol. After vacuum drying, samples were reconstituted and mass spectrometrically analyzed as described above, with the exception of shortening the elution gradient to 90 min. Fragmentation was performed using the HCD cell of the Orbitrap mass analyzer as mentioned earlier [55].

Database searches were performed using MaxQuant software (version 1.6.3.4; https://www.maxquant.org/; accessed on 18 July 2023) [56]. For peptide identification and quantitation, MS/MS spectra were correlated with the UniProt human reference proteome set (https://www.uniprot.org/; accessed on 18 July 2023), employing the built-in Andromeda search engine [57]. The respective TMT modifications and carbamidomethylated cysteine were considered as fixed modifications along with oxidation (M), and acetylated protein N-termini as a variable modification. False discovery rates were set on both the peptide and protein level to 0.01. Subsequent data analysis was performed employing MS Excel and GraphPad PRISM software (version 9.5.0; GraphPad Software Inc., Boston, MA, USA). For outlier analysis on TMT datasets, significance B was calculated using Perseus (https://maxquant.org/perseus/; accessed on 18 July 2023). For label-free analysis, a cutoff ratio was employed, and for visualization of proteins exclusive to either medium, fold change was set to log2 = 5.

Pathway and process enrichment analysis was performed on proteins highly expressed by cells of media 1 and 13 separately using Metascape software (version v3.5.20230501) [58]. To this end, the following parameters were used: *p*-value < 0.01, a minimum count of 3, and an enrichment factor >1.5. For interaction network visualization, Cytoscape software (version 3.7.1) [59] was employed using interaction data retrieved on the set of regulated proteins via https://www.string-db.org/ (accessed on 18 July 2023) [60].

### 2.4. Characterization of Media and Conditioned Media

#### 2.4.1. Magnetic-Bead-Based Multiplex Analyses

Different factors were analyzed in samples of media and conditioned media (time point of harvest) for cells grown in media 1, 4, 10, 7, and 13 by using a magnetic-bead-based multiplex analysis technology (Merck KGaA, Darmstadt, Germany). The following analytes were included: angiopoietin-2, Dickkopf-related protein 1 (DKK1), epidermal growth factor (EGF), endoglin, FGF-2, FGF-23, follistatin, fractalkine, growth-regulated oncogene α (GROα) or (C-X-C motif) ligand (CXCL)1, heparin-binding EGF-like growth factor (HB-EGF), hepatocyte growth factor (HGF), IGF-I, IGF-II, interleukin (IL)-6, IL-8, insulin, leptin, monocyte chemoattractant protein (MCP)-1 or (C-C motif) ligand (CCL)2, MCP-3 or CCL7, macrophage colony-stimulating factor (M-CSF), monokine induced by interferon γ (IFNγ) (MIG) or CXCL9, matrix metalloproteinase (MMP)-1, MMP-2, MMP-7, MMP-9, MMP-10, osteocalcin (OC), osteoprotegerin (OPG), osteopontin (OPN), PDGF-AA, PDGF-AB/BB, placental growth factor (PlGF), parathyroid hormone (PTH), regulated and normal T-cell-expressed and secreted (RANTES) or CCL5, soluble CD40 ligand (sCD40L), sclerostin (SOST), TGFβ, tumor necrosis factor (TNF)β, thrombospondin-1 (TSP-1), vascular endothelial growth factor (VEGF)-A and VEGF-C. Briefly, samples were thawed and the analyses were run in duplicates (n = 2) in 96-well plates as per the manufacturer’s instructions (changes were obtained for the analysis of IGF-I and IGF-II, where a lower dilution was used for the neutralization step, and regarding the matrix solution of all assays, where assay buffer was used). Samples were diluted with sample diluent for analysis of TGFβ (1:3) or αMEM without supplements for analysis of insulin (1:100; only for approaches of media 4, 7, 10, 13), RANTES (1:50), and TSP-1 (1:20). Only wells with bead counts ≥35 were included in the analyses (except for analytes VEGF-C, MMP-7 and MIG). Concentrations below detection limits were assumed to be zero. Consumption and production of factors were calculated by the difference in respective concentrations in media and conditioned media. Both parameters were normalized to one million harvested cells and a time interval of one day. With regard to calculations for consumption and production, two scenarios have to be generally noted. First, it cannot be excluded that concentrations of factors decreased in conditioned media for reasons other than consumption by cells. Second, cells may have consumed but also produced factors in similar amounts, thereby resulting in no consumption as per our definition. Analytes that were measured but excluded from any analysis due to concentrations below 10 pg/mL included adrenocorticotropic hormone (ACTH), bone morphogenetic protein 9 (BMP-9), endothelin-1, FGF-1, IFNγ, IL-1α, IL-1β, IL-1 receptor antagonist (IL-1RA), IL-2, IL-10, IFNγ induced protein 10 (IP-10) or CXCL10, macrophage inflammatory protein (MIP)-1β or CCL4, TGFα, TNFα, and VEGF-D.

#### 2.4.2. TNF-Inducible Gene 6 (TSG-6) Enzyme-Linked Immune Sorbent Assay (ELISA)

TSG-6 was analyzed in samples of media and conditioned media (time point of harvest) for cells grown in media 1, 4, 10, 7, and 13 by ELISA. Recombinant human TSG-6 (R&D Systems, Inc.) was used as standard. The assay was run in duplicate (n = 2) in 96-well plates as follows. Plates were coated with anti-TSG-6 antibody (clone A38.1.20; Santa Cruz Biotechnology, Inc., Dallas, TX, USA) at 10 µg/mL (in phosphate-buffered saline (PBS)) overnight at 4 °C. All following steps were performed at RT. Wells were washed four times with wash buffer (Quantikine ELISA Wash Buffer 1 1:25 in distilled water (aqua dest); R&D Systems, Inc.) with 1 min soak time in between. Blocking of wells was performed by the addition of blocking buffer (Reagent Diluent Concentrate 2 (R&D Systems, Inc.) 1:10 in aqua dest with 0.05% Tween^®^20 (Sigma-Aldrich Chemie GmbH)) and incubation for 1 h. Wells were washed as described above. A 1:2 dilution series of the standard stock solution was performed with blocking buffer resulting in standards ranging from 4.800 pg/mL to 75 pg/mL. Blocking buffer was used as 0 pg/mL standard (background). Standards and thawed samples were incubated on the plate for 2 h with agitation. Wells were washed as described above. A biotinylated anti-TSG-6 antibody (polyclonal; R&D Systems, Inc.) was added at 0.5 µg/mL (in blocking buffer) and incubated for 2 h with agitation for detection of TSG-6. Wells were washed as described above. Streptavidin coupled to horseradish peroxidase (HRP) (Streptavidin-HRP 1:200 in blocking buffer; R&D Systems, Inc.) was added and incubated for 20 min in the dark. Wells were washed as described above. HRP substrate (R&D Systems, Inc.) was added and incubated for 30 min in the dark prior to the addition of stop solution (Stop Solution 2N Sulfuric Acid; R&D Systems, Inc.). Absorption was measured at 450 nm and 570 nm (reference wavelength) using the microplate reader POLARstar Omega (BMG LABTECH GmbH, Ortenberg, Germany) with Reader Control (version 5.70 R2) and MARS Data Analysis software (version 4.00 R2), respectively.

### 2.5. Statistics

Statistical analysis was performed with GraphPad PRISM software (version 9.3.1; Graphpad Software Inc.). At least three independent experiments with MSCs of three different donors (N ≥ 3) were carried out (except for analysis of MIG (all media) and IGF-II (media 10), which were excluded from further statistical analysis as well as proteomic analyses). Data are presented as mean ± standard deviation (SD). Data were tested for normal distribution using a Shapiro–Wilk normality test. Significant differences between groups were investigated as follows. All groups were tested against the control group medium 1. Paired test methods were used for all analyses except for flow cytometry. In the case of normal distribution, one-way analysis of variance (ANOVA) was chosen for data sets with no missing values, and mixed-effects analysis was used for data with missing values. Geisser-Greenhouse correction was applied for both. If there was no normal distribution, a Friedman test was used for data sets with no missing values, and a Kruskal–Wallis test was applied for data sets with missing values. Due to many missing values, unpaired test methods were used for flow cytometric analyses. Homogeneous variance was tested by a Brown–Forsythe test. One-way ANOVA was used for normally distributed data sets and in case of inhomogeneous variance, Welch correction was applied. If there was no normal distribution, a Kruskal–Wallis test was used. For all analyses, Holm–Šídák’s, Dunnet’s, Dunnet’s T3, or Dunn’s methods were applied for correction of multiple testing.

## 3. Results

### 3.1. Proliferation of Cells Can Be Increased by Media Containing at Least 50% StemMACS^TM^

**Figure 1:**
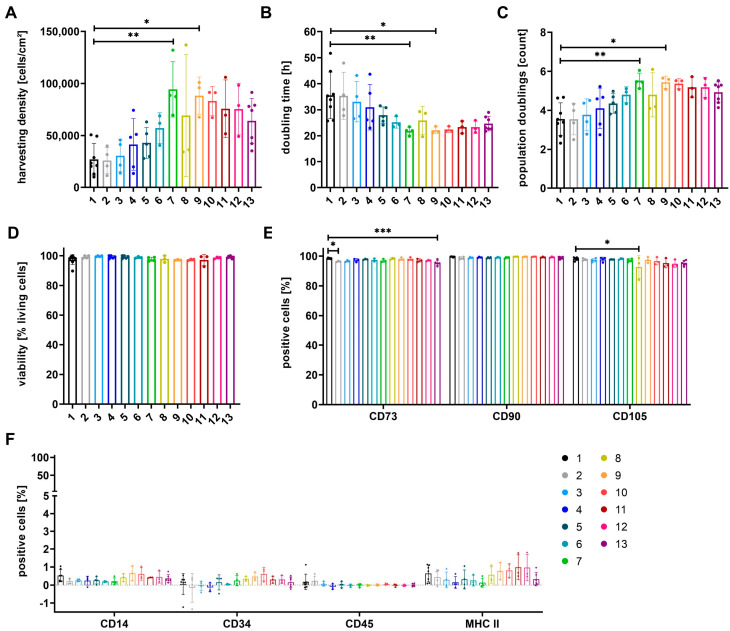
Expansion parameters and basic characterization of MSCs grown in media 1 to 13. MSCs, primarily isolated in medium αMEM+8%PL, were expanded in media 1 to 13 (for composition see Table 1; colors as indicated in scheme) for P3. The expansion parameters harvesting density (**A**), doubling time (**B**), and number of population doublings (**C**) were determined in addition to the viability of cells (**D**). The expression of identity markers (CD73, CD90, and CD105) (**E**) and purity markers (CD14, CD34, CD45, and MHC II) (**F**) was analyzed by flow cytometry. Data are presented as mean ± SD and N ≥ 3 independent experiments were performed. Statistically significant differences are depicted as follows: *: *p* < 0.05; **: *p* < 0.01; ***: *p* < 0.001.

MSCs, primarily isolated in αMEM+8%PL, were expanded in 13 different media for P3. Media were composed of various ratios of the XF/PL medium αMEM+8%PL (medium 1) and SF/XF StemMACS^TM^ (medium 13) as illustrated in Table 1. Proliferation of cells was compared by analysis of different expansion parameters (harvesting density, doubling time, number of population doublings). In addition, the viability of cells and expression of identity and purity markers were investigated (Figure 1).

Proliferation of cells was increased for those grown in an SF/XF medium (medium 13) in comparison to cells expanded in an XF/PL medium (medium 1), as shown by higher harvesting density, the number of population doublings, and a reduced doubling time (Figure 1A–C). Proliferation could be further enhanced by the culture of cells in mixtures of both media containing at least 50% StemMACS^TM^ (media 8–12). Significantly higher harvesting densities and numbers of population doublings, as well as significantly reduced doubling times, were observed for cells grown in media 7 and 9 (Figure 1A–C). The viability of cells showed no significant differences between the different media (Figure 1D). Significantly lower expression of CD73 was obtained for cells grown in media 2 and 13 in addition to a significantly reduced expression of CD105 for cells grown in medium 8. In contrast, no significant changes were obtained for the expression of CD90 and purity markers CD14, CD34, CD45, and MHC II (Figure 1E,F).

After the analysis of the expansion of MSCs in several ratios of αMEM+8%PL and StemMACS^TM^, the following experiments were performed with the pure media (media 1+13) and the media mixtures 4, 7, and 10.

**Figure 2:**
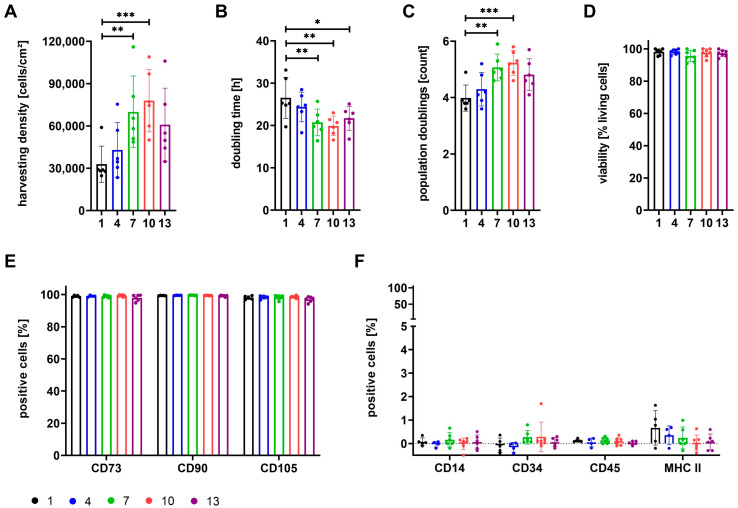
Expansion parameters and basic characterization of MSCs grown in media 1, 4, 7, 10, and 13. MSCs were isolated and subsequently expanded in media 1 (100% αMEM+8%PL; black), 4 (95% αMEM+8%PL + 5% StemMACS^TM^; blue), 7 (50% αMEM+8%PL + 50% StemMACS^TM^; green), 10 (5% αMEM+8%PL + 95% StemMACS^TM^; red) and 13 (100% StemMACS^TM^; violet) for P1. The expansion parameters harvesting density (**A**), doubling time (**B**), and number of population doublings (**C**) were determined in addition to the viability of cells (**D**). The expression of identity markers (CD73, CD90, and CD105) (**E**) and purity markers (CD14, CD34, CD45, and MHC II) (**F**) was analyzed by flow cytometry. Data are presented as mean ± SD and N ≥ 4 independent experiments were performed. Statistically significant differences are depicted as follows: *: *p* < 0.05; **: *p* < 0.01; ***: *p* < 0.001.

The proliferation of cells in P1 was compared for cells already isolated in the respective media during P0. Expansion parameters, viability of cells, and expression of identity and purity markers are illustrated in Figure 2.

Cells isolated and expanded in media containing any ratio of StemMACS^TM^ (media 4+7+10+13) showed increased proliferation as compared to cells grown in αMEM+8%PL (medium 1). Harvesting density and number of population doublings were significantly higher for cells cultured in media 7 and 10, and doubling time was significantly reduced for cells grown in media 7, 10, and 13 (Figure 2A–C). Media mixtures containing at least 50% StemMACS^TM^ as well as PL (media 7+10) further enhanced cell proliferation as compared to growth in StemMACS^TM^ alone (medium 13). No significant differences between cells were obtained for viability (Figure 2D) or the expression of identity and purity markers (Figure 2E,F).

Regardless of whether the MSCs were already cultured in media with the respective supplement from P0 (Figure 2) or were only placed in the respective media in P3 (Figure 1), we observed the same effects of the supplements on the expansion parameters, viability, and expression of surface antigens.

### 3.2. Proteomic Analyses Indicate Differences for Growth Media and Respective Cells

**Figure 3:**
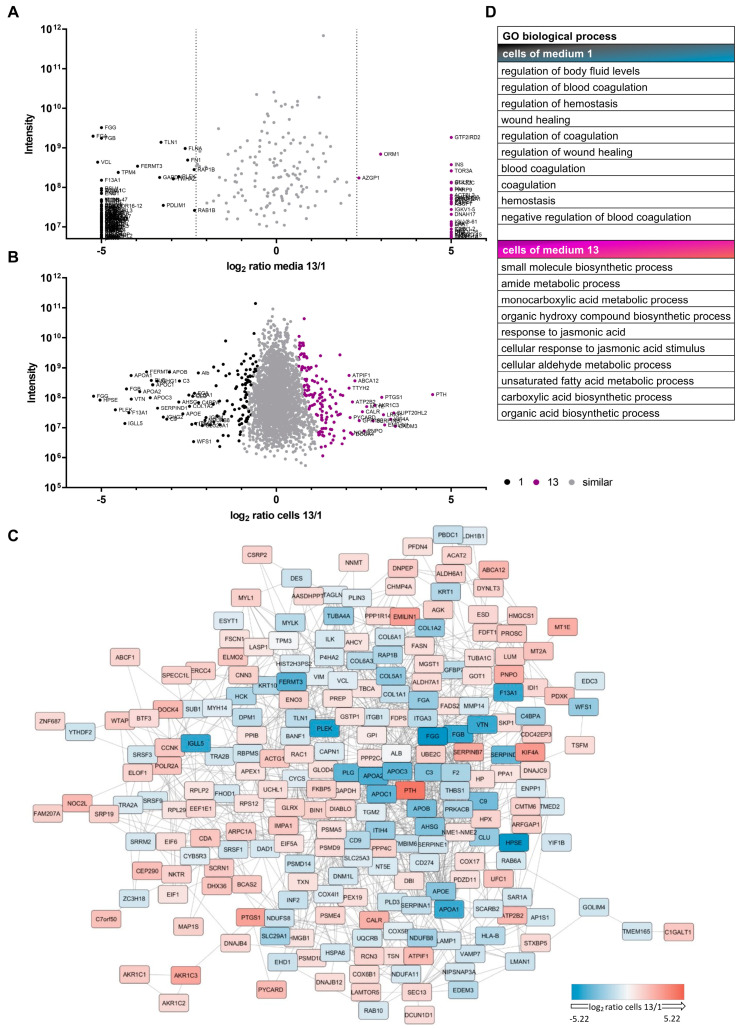
Proteomic analyses of media and MSCs. MSCs, isolated and expanded in αMEM+8%PL (medium 1; black) or StemMACS^TM^ (medium 13; violet), and respective media were used for proteomic analyses. Log2 ratio of proteins identified in media 1 and 13 (**A**) or expressed by cells grown in media 1 and 13 (**B**) are illustrated. Proteins with similar quantity or expression are shown as gray dots, proteins with high quantity in medium 1 (**A**) or highly expressed in cells grown in medium 1 (**B**) are shown as black dots and those with high quantity in medium 13 (**A**) or high expression in cells grown in medium 13 (**B**) are shown as violet dots, respectively. (**C**) Differentially expressed proteins shown in (**B**) were queried for known interactions on StringDb [60] and visualized. (**D**) Expressed proteins seem to play a role in various biological processes based on gene ontology (GO) enrichment analysis.

Due to differential proliferative capacities for cells grown in αMEM+8%PL (medium 1) and StemMACS^TM^ (medium 13), proteomic analyses were performed for one batch of growth media as well as respective cells from one representative MSC donor in order to have an indication about altered cellular processes (Figure 3 and Figure S1, Tables S4 and S5).

Proteomic analyses indicated differential expression of proteins for cells grown in media 1 and 13 (Figure 3A and Table S4) in addition to a variable quantity of several proteins in respective media, where some were exclusively present in only one of the media (Figure 3B and Table S5). Differentially expressed proteins by cells grown in media 1 and 13 formed a complex proteomic network, and clusters were present for cells cultured in each media (Figure 3C). This clustering of proteins could be supported by pathway and process enrichment analyses, which suggested involvement in different biological processes (Figure 3D and Figure S1), cellular components, and molecular functions (Figure S1) for those cells. The resulting gene ontology (GO) terms indicated, amongst others, differences in particular proteins or respective genes involved in wound healing, coagulation, hemostasis, and binding of PDGF for MSCs grown in XF/PL conditions or metabolic processes of, e.g., growth factors, fatty acids, and hormones for MSCs grown in SF/XF media (Figure S1). The observed indications were used as the basis for further experiments, where alterations in cell characteristics as a result of different growth media should be confirmed by various additional analyses.

### 3.3. Cells Show Different Basic Metabolism Depending on Growth Media

**Figure 4:**
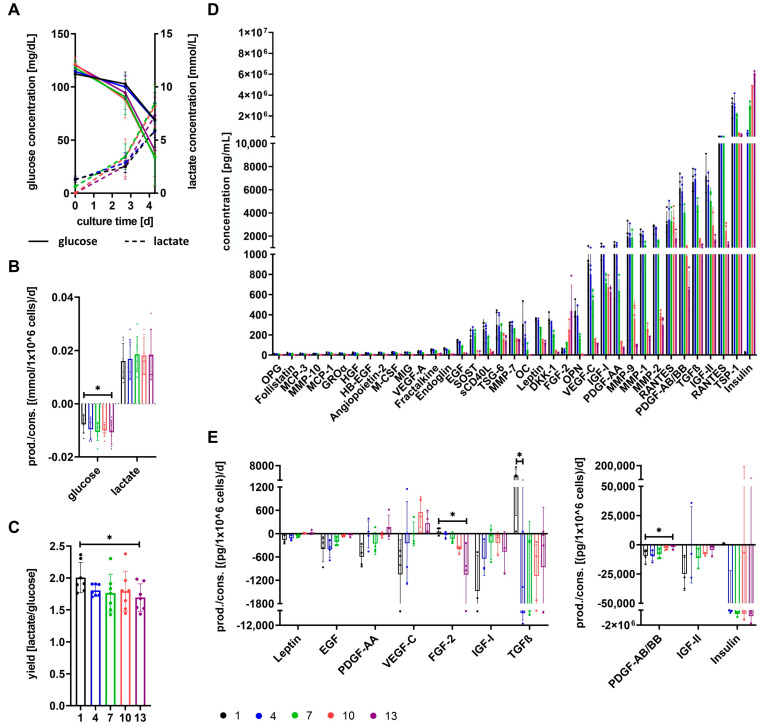
Consumption and production of metabolic factors by MSCs. MSCs were grown in media 1 (100% αMEM+8%PL; black), 4 (95% αMEM+8%PL + 5% StemMACS^TM^; blue), 7 (50% αMEM+8%PL + 50% StemMACS^TM^; green), 10 (5% αMEM+8%PL + 95% StemMACS^TM^; red) and 13 (100% StemMACS^TM^; violet). (**A**) Glucose and lactate concentrations were determined in media at the beginning of the cell cultures (d0) and in conditioned media at the time point of media exchange (d2–3) and harvesting of cells (d4–6). Glucose consumption and lactate production of one million cells per day (**B**) and the yield of lactate per glucose (**C**) were analyzed for the time between media exchange and harvesting of cells. (**D**) Concentrations of the factors OPG, follistatin, MCP-3, MMP-10, MCP-1, GROα, HGF, HB-EGF, angiopoietin-2, M-CSF, MIG, VEGF-A, fractalkine, endoglin, EGF, SOST, sCD40L, TSG-6, MMP-7, OC, leptin, DKK1, FGF-2, OPN, VEGF-C, IGF-I, PDGF-AA, MMP-9, MMP-1, MMP-2, RANTES, PDGF-AB/BB, TGFβ, IGF-II, TSP-1, and insulin were analyzed in media. (**E**) Consumption and production of growth factors EGF, PDGF-AA, VEGF-C, FGF-2, IGF-I, TGFβ, PDGF-AB/BB and IGF-II, as well as hormones leptin and insulin, was calculated between media exchange and harvesting of cells and normalized to 1 × 10^6^ MSC/24 h. Data are presented as mean ± SD and N ≥ 3 independent experiments were performed (except for analysis of MIG (all media) and IGF-II (medium 10)). Statistically significant differences are depicted as follows: *: *p* < 0.05.

Based on differences for growth media αMEM+8%PL (medium 1) and StemMACS^TM^ (medium 13), as well as respective cells indicated by proteomic analyses, metabolic basics were further analyzed for cells grown in media 1, 4, 7, 10, and 13 (Figure 4).

Glucose concentrations decreased and lactate concentrations increased in conditioned media over culture time for cultures of all media (Figure 4A). Glucose was consumed by the cells, whereas lactate was produced as shown in Figure 4B. Glucose consumption and accordingly lactate production were lowest for cells grown in medium 1, and a significantly lower glucose consumption was obtained compared to cells cultured in medium 13 (Figure 4B). The yield of lactate per glucose decreased with increasing content of StemMACS^TM^ in culture media and was significantly reduced for cells cultured in medium 13 (Figure 4C). The factors OPG, follistatin, MCP-3, MMP-10, MCP-1, GROα, HGF, HB-EGF, angiopoietin-2, M-CSF, MIG, VEGF-A, fractalkine, endoglin, EGF, SOST, sCD40L, TSG-6, MMP-7, OC, leptin, DKK1, FGF-2, OPN, VEGF-C, IGF-I, PDGFA-AA, MMP-9, MMP-1, MMP-2, RANTES, PDGF-AB/BB, TGFβ, IGF-II, TSP-1, and insulin were identified at different concentrations in media. Only FGF-2 and insulin were present at the highest concentrations in medium 13, whereas all of the other factors showed the highest concentrations in medium 1 (Figure 4D and Figure S2). In the next step, we measured the change in growth factors and hormones during the culture of MSCs in different media. The factors leptin, PDGF-AA, VEGF-C, IGF-I, and IGF-II were consumed most by cells grown in medium 1, and the factors EGF and PDGF-AB/BB showed equally highest consumption by cells cultured in media 1 and 4. Except for IGF-I, which showed the lowest consumption by cells cultured in media 7 and 10, consumption of all other aforementioned factors (leptin, EGF, PDGF-AB/BB, and IGF-II) was lowest for cells cultured in medium 13. Consumption of PDGF-AB/BB was significantly reduced for cultures in medium 13 compared to medium 1. In contrast, FGF-2 and insulin showed the highest consumption for cells grown in medium 13 and lowest for cells grown in medium 1, resulting in a significant difference for FGF-2. TGFβ was consumed most by cells cultured in medium 4, whereas MSCs grown in medium 1 produced TGFβ, resulting in a significant difference between medium 1 and medium 4 (Figure 4E). Other growth factors (FGF-23, HB-EGF, HGF, PlGF, and VEGF-A) were not consumed by cells cultured in any media. However, it has to be generally noted that it cannot be excluded that the concentration of factors decreased in conditioned media for reasons other than consumption by cells.

### 3.4. Expression of Surface Antigens Shows Alterations between Cells Grown in Different Media

**Figure 5:**
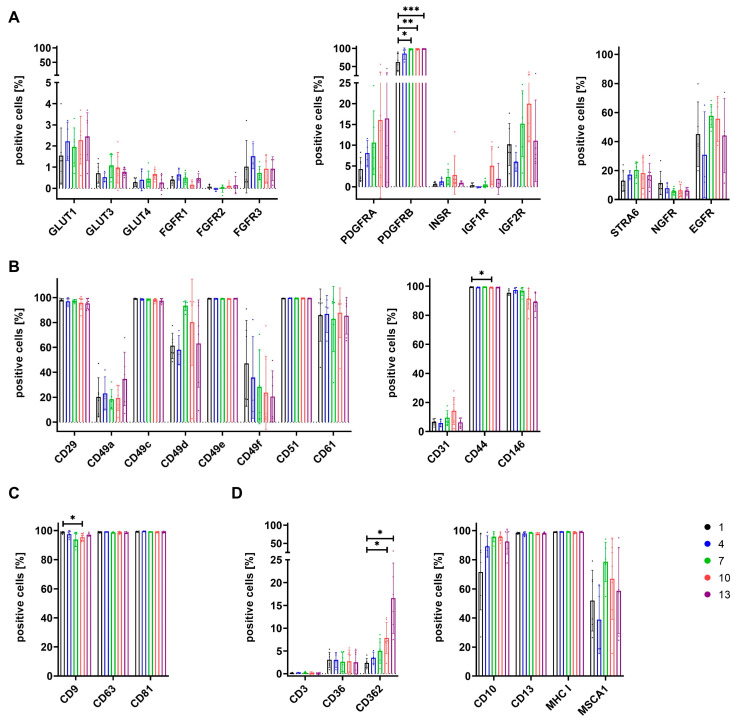
Surface antigen expression by MSCs grown in media 1, 4, 7, 10, and 13. MSCs grown in media 1 (100% αMEM+8%PL; black), 4 (95% αMEM+8%PL + 5% StemMACS^TM^; blue), 7 (50% αMEM+8%PL + 50% StemMACS^TM^; green), 10 (5% αMEM+8%PL + 95% StemMACS^TM^; red), and 13 (100% StemMACS^TM^; violet) were analyzed for the expression of different surface antigens. These included metabolism-related markers GLUT1, GLUT3, GLUT4, FGFR1, FGFR2, FGFR3, PDGFRA, PDGFRB, INSR, IGF1R, IGF2R, STRA6, NGFR, and EGFR (**A**), cell adhesion-related markers CD29, CD49a, CD49c, CD49d, CD49e, CD49f, CD51, CD61, CD31, CD44, and CD146 (**B**), tetraspanins CD9, CD63, and CD81 (**C**), as well as the additional markers CD3, CD36, CD362, CD10, CD13, MHC I, and MSCA1 (**D**). Data are presented as mean ± SD and N ≥ 3 independent experiments were performed. Statistically significant differences are depicted as follows: *: *p* < 0.05; **: *p* < 0.01; ***: *p* < 0.001.

Cells grown in media 1, 4, 7, 10, and 13 were further characterized by the analysis of the expression of various surface antigens. The selection of the markers was informed by the results of the proteomic analysis. These included metabolism-related markers, cell adhesion-related markers, tetraspanins involved in the regulation of vesicles, and some additional markers summarized in Figure 5.

A low expression of glucose transporters GLUT1, GLUT3, and GLUT4, as well as FGF receptors FGFR1, FGFR2, and FGFR3, was observed for cells grown in all media (Figure 5A). PDGFRA showed a low to moderate expression that increased for cells grown in media with a higher percentage of SF/XF media (StemMACS^TM^). Similarly, expression of PDGFRB also raised with increasing content of SX/XF media and was significantly increased for cells grown in media 7, 10, and 13 (Figure 5A). Receptors for insulin and IGF-I were only marginally expressed by cells of all media, whereas increased expression was observed for IGF2R, and expression levels for both IGF receptors were highest for cells cultured in medium 10 (Figure 5A). Low to moderate expression was obtained for STRA6 and NGFR irrespective of growth media. EGFR expression of cells differed between growth media but also between single MSC donors within one group. The highest expression levels and lowest variation between MSC donors were observed for cells grown in medium 7 (Figure 5A). The integrins CD29, CD49c, CD49e, and CD51 showed high expression of about 100% positive cells for all media. CD49a and CD49f were only expressed at low to moderate levels, where CD49f expression decreased with increasing content of StemMACS^TM^ in media. Expression of CD49d and CD61 was moderate to high, and especially cells grown in media 7 showed high expression of CD49d (Figure 5B). Regarding cell adhesion molecules, expression of CD31 was low to moderate, and high expression levels of CD44 were obtained for cells of all media, though a significant difference was observed between cells grown in media 1 and 10. CD146 expression was also high for cells of all media, but showed a slight reduction for cells cultured in media 10 and 13 (Figure 5B). The tetraspanins CD9, CD63, and CD81 were expressed at high levels for cells grown in all media. Only CD9 showed a significantly lower expression for cells in medium 10 (Figure 5C). With regard to some additionally analyzed surface antigens, a low expression below 5% positive cells was obtained for CD3 and CD36 for cells of all media. CD362 expression increased with the percentage of StemMACS^TM^ in media and was significantly increased for cells grown in media 10 and 13 (Figure 5D). CD10 was expressed at high levels for cells of all media except for a slightly reduced expression for those of medium 1, and high expression was observed for all cells regarding CD13 and MHC I. MSCA1 expression varied between cells grown in different media, but also between MSC donors of the same media. The lowest expression was obtained for cells grown in medium 4 and the highest expression was obtained for those grown in medium 7 (Figure 5D).

### 3.5. The Secretome of Cells Varies after Expansion in Different Growth Media

The secretome of cells grown in media 1, 4, 7, 10, and 13 was investigated for several functionally relevant factors.

**Figure 6:**
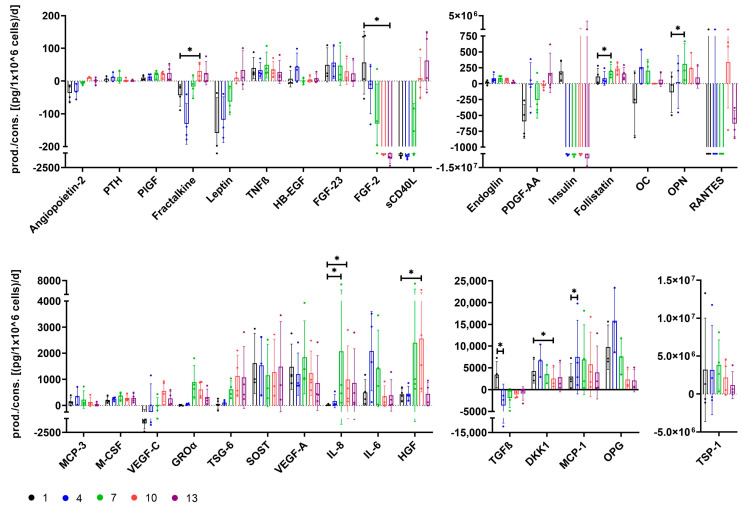
Secretion of functional factors by MSCs grown in media 1, 4, 7, 10, and 13. MSCs were grown in media 1 (100% αMEM+8%PL; black), 4 (95% αMEM+8%PL + 5% StemMACS^TM^; blue), 7 (50% αMEM+8%PL + 50% StemMACS^TM^; green), 10 (5% αMEM+8%PL + 95% StemMACS^TM^; red) and 13 (100% StemMACS^TM^; violet). The secretion of factors angiopoietin-2, PTH, PlGF, fractalkine, leptin, TNFβ, HB-EGF, FGF-23, FGF-2, sCD40L, endoglin, PDGF-AA, insulin, follistatin, OC, OPN, RANTES, MCP-3, M-CSF, VEGF-C, GROα, TSG-6, SOST, VEGF-A, IL-8, IL-6, HGF, TGFβ, DKK1, MCP-1, OPG and TSP-1 was analyzed for the period between media exchange and harvesting of cells and normalized to 1 × 10^6^ MSC/24 h. *: *p* < 0.05.

Only cells grown in medium 1 showed secretion of factors FGF-2, insulin, and TGFβ, and secretion of FGF-2 and TGFβ was significantly reduced for cells cultured in media 13 and 4, respectively (Figure 6). HB-EGF was only secreted by cells grown in medium 4, and the highest secretion levels for those cells were also obtained for factors MCP-3, DKK,1 and OPG. The latter factors were also secreted at reduced levels by cells grown in the other media, though OPG secretion was especially low for cells grown in media 10 and 13, and DKK1 secretion was significantly reduced for cells grown in medium 10 (Figure 6). Secretion of follistatin was achieved for cells of all media, but secretion was significantly increased for those grown in medium 7 compared to medium 1 (Figure 6). RANTES and angiopoietin-2 secretion could not be identified for cells of any other media than 10; yet, secretion levels were also low for those cells (Figure 6). Secretion of leptin, sCD40L, and PDGF-AA was only achieved for cells cultured in medium 13 (Figure 6). OC and IL-6 were secreted most by cells grown in media 4 and 7. Endoglin and MCP-1 secretion was increased for cells grown in all media mixtures (media 4+7+10), with significantly higher MCP-1 secretion for cells grown in medium 4 (Figure 6). HGF secretion was elevated for cells cultured in medium 7 and even significantly for those grown in medium 10 (Figure 6). OPN, GROα, TSG-6, and IL-8 secretion was increased for cells grown in media 7, 10, and 13. Cells cultured in medium 1 showed almost no secretion of these factors at all, leading to a significantly lower secretion of OPN compared to cells grown in medium 7 and of IL-8 compared to those grown in media 7 and 10 (Figure 6). Only cells grown in media with high percentages of StemMACS^TM^ (media 10+13) secreted fractalkine and VEGF-C (Figure 6). General trends of higher secretion with a high content of SF/XF media were observed for factors PlGF, leptin, sCD40L, and TSG-6, whereas trends of lower secretion were obtained for factors FGF-23, FGF-2, insulin, VEGF-A, and TSP-1 (Figure 6). Secretion of factors PTH, TNFβ, M-CSF, and SOST was comparable between cells grown in different media (Figure 6).

### 3.6. Differentiation Potential of Cells Depends on Growth Media

**Figure 7:**
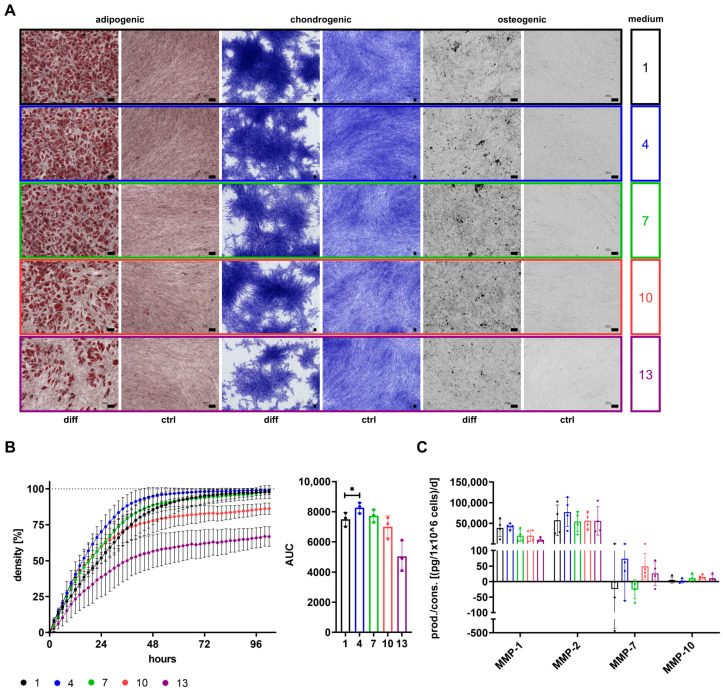
Differentiation and migration potential of MSCs grown in media 1, 4, 7, 10, and 13. MSCs were expanded in media 1 (100% αMEM+8%PL; black), 4 (95% αMEM+8%PL + 5% StemMACS^TM^; blue), 7 (50% αMEM+8%PL + 50% StemMACS^TM^; green), 10 (5% αMEM+8%PL + 95% StemMACS^TM^; red) and 13 (100% StemMACS^TM^; violet) and analyzed for their differentiation potential and migratory capacity. (**A**) MSCs were differentiated into cells of adipogenic, chondrogenic, and osteogenic lineages by culture in specific differentiation media (diff). Control cells were expanded in αMEM+20%FCS (ctrl). Cells of adipogenic differentiation were stained by Oil Red O and hematoxylin. Methylene blue staining was performed to detect chondrogenic differentiation. Activity of alkaline phosphatase was visualized by 5-bromo-4-chloro-3-indolylphosphate (BCIP)/nitroblue tetrazolium (NBT) substrate to detect osteogenic differentiation. Pictures of cells were taken by an inverted phase contrast microscope with 4 times (chondrogenic) and 10 times (adipogenic and osteogenic) magnification, respectively. Black scale bars indicate 100 µm. (**B**) Migratory potential of cells was investigated by a scratch wound assay. For this, cells were grown in media 1, 4, 7, 10, and 13 in a 96-well plate until confluence of cell cultures was reached. A scratch wound area was created into the cell layer and migration of cells was analyzed for 96 h. Relative cell density was identified by IncuCyte^®^ S3 Live-Cell Analysis system. The area under the curve (AUC) was determined for observed analyses curves of wound density over time. (**C**) The secretion of MMP-1, MMP-2, MMP-7, and MMP-10 was analyzed for cells grown in media 1, 4, 7, 10, and 13. Data are presented as mean ± SD and N = 2 (A) or N ≥ 3 (**B**,**C**) independent experiments were performed. Statistically significant differences are depicted as follows: *: *p* < 0.05. Representative images were chosen for differentiation assays.

The differentiation potential is a known functional property of MSCs and was therefore investigated for cells expanded in media 1, 4, 7, 10, and 13 (Figure 7).

Cells grown in media 1, 4, 7, 10, and 13 could be differentiated into cells of adipogenic, chondrogenic, and osteogenic lineages. The adipogenic differentiation seemed to be decreasing with an increasing percentage of StemMACS^TM^ in media, while chondrogenic and osteogenic differentiation appeared to be only less distinct for cells grown in medium 13 (Figure 7A). However, differentiation potential was only evaluated on a visual basis.

### 3.7. SF/XF Culture Conditions Reduce Migratory Potential of MSCs

Besides differentiation potential, migratory capacity is an important functional feature of MSCs. Migration of cells and secretion of different MMPs was analyzed for cells grown in media 1, 4, 7, 10, and 13 (Figure 7B,C). The highest migratory capacity was observed for cells grown in medium 4, as shown by a significantly higher AUC followed by cells cultured in media 1 and 7. Cells grown in media 10 and 13 were not capable of reaching 100% cell density in the wound area (Figure 7B). The highest secretion of MMPs was observed regarding MMP-1 and MMP-2 that were secreted by cells of all media. Reduced secretion of MMP-1 was obtained for cells of media with high content of StemMACS^TM^ (media 7+10+13), while secretion of MMP-2 was enhanced for cells grown in medium 4. Secretion of MMP-7 did not only vary between cells of different media but also between MSC donors, and was only secreted by cells grown in media 4, 10, and 13. MMP-10 was only secreted at very low levels for cells of all media (Figure 7C).

## 4. Discussion

Therapeutic application of MSCs implies their ex vivo expansion in appropriate expansion media which can strongly affect MSC characteristics [61]. For this, human-derived PL has been established as a safe and XF-growth-promoting supplement in cell culture [34,35,36]. However, it contains a plethora of components, and knowledge regarding which of these many components is essential for its biological activity is limited. This is a major drawback in its use for GMP-grade expansion, which aims for the most defined culture conditions possible. As an alternative, many defined SF/XF expansion media were developed in recent years, though some of them resulted in inefficient cell growth [46,47,48]. The use of a standardized, chemically defined medium by itself is not necessarily an advantage. It is also necessary to understand the effects of these media on the phenotype and function of the MSCs expanded in them. In order to investigate the impact of growth media on MSCs, we did not only culture the cells in either XF/PL (αMEM+8%PL; medium 1) or SF/XF (StemMACS^TM^; medium 13) media, but also in mixtures of both, which may overcome the aforementioned disadvantages of each group. The potential of each media in priming for specific therapeutic applications was evaluated by analysis of several cell characteristics.

Expansion of MSCs that have been previously isolated following our standard protocol in αMEM+8%PL was compared for 13 different media in order to obtain primary information on mixtures that are suitable for MSC expansion. Based on these results, further analyses were reduced to media 1, 4, 7, 10, and 13, i.e., the pure form of each medium, a 50:50 mix, and two mixtures with a small proportion (5%) of the other medium. MSCs used for these analyses were already isolated from BM and subsequently expanded in respective media. Proliferation was generally increased for cells grown in 100% SF/XF media (StemMACS^TM^; medium 13) as compared to those expanded in 100% XF/PL media (αMEM+8%PL; medium 1) and proliferation could be even significantly enhanced by a mixture of both media in specific ratios (media 7+9+10). Thus, a combination of SF/XF media with components contained only in PL seems to bring about the best ex vivo expansion of MSCs. Given this increased promotion of cell growth by a mixture of media, two main hypotheses came up. On the one hand, either medium could potentially include important factors completely lacking in the other media or only being present in insufficient amounts. On the other hand, there might be also factors impeding cell growth whose concentrations could be reduced by a mixture with the other media.

A general trend of decreasing expression of CD73 and CD105 by cells grown with a higher content of StemMACS^TM^ was observed during the first experiments using cells initially isolated in αMEM+8%PL and afterward expanded in media 1 to 13. A reduction in CD73 and CD105 expression was also described by Lensch et al. for cells grown in StemMACS^TM^, though expression levels could be restored by expansion time [62]. Similarly, no impact on the expression of identity and purity markers was observed when cells were exclusively cultured in media 1, 4, 7, 10, and 13 (that is, isolation and expansion of cells in either medium), assuming a stress reaction of cells after a change in media during the first experiments.

We assumed that differences in metabolism were responsible for the altered proliferation of cells and for divergent cell characteristics observed throughout the study. In order to investigate these hypotheses, proteomic analyses of basic media αMEM+8%PL and StemMACS^TM^, as well as respective cells grown in these media, were performed as a first step. The results indicated substantial differences between these prototypic media. Also, MSCs grown in these media showed differential expression of proteins that were involved in several cellular processes. MSC growth in PL is rather associated with a profile linked to the regulation of wound healing and coagulation. In contrast, a culture of MSCs in the SF/XF medium StemMACS^TM^ seems to be associated with a change in metabolic aspects (biosynthesis of small molecules and metabolism of aldehyde, amide, unsaturated fatty acid, carboxylic acid). Furthermore, differences between cells of media 1 and 13 were also apparent for the binding of PDGF.

We analyzed the consumption and/or production of glucose and lactate and several other factors during MSC cultures. Investigation of glucose and lactate metabolism revealed significant differences between cells grown in media 1 and 13. The higher the percentage of αMEM+8%PL, the higher the yield of lactate per glucose, indicating a higher proportion of inefficient energy production by glycolysis as discussed by Schop et al. [53].

Analysis of growth factors, cytokines, chemokines, hormones, and other factors in media showed higher concentrations for αMEM+8%PL-based media except for FGF-2 and insulin, the latter being totally absent in αMEM+8%PL. TGFβ, FGF-2, and PDGF-BB were identified as essential components for MSC proliferation [35,63], and TGFβ and PDGF-BB were only available at low concentrations during SF/XF conditions. The highest consumption of TGFß was observed for cells grown in medium 4, whereas those in medium 1 showed no consumption at all, but even secretion. TGFβ can not only appear in its active form but also in an inactive form where it is bound to latency-associated peptides. Since only cells grown in medium 1 showed no TGFβ consumption at all, it may be the case that TGFβ could not be used by these cells due to its presence only in an inactivated form, thereby leading to lower proliferation. TGFβ has been shown to be activated by several different mechanisms including shifts in pH or temperature, reactive oxygen species, TSP-1, deglycosylation, proteases, or other factors like retinoic acid, sex hormones, vitamin D, and MMPs [64,65]. Factors included in the media StemMACS^TM^ may have resulted in the activation of TGFβ in media 4, 7, 10, and 13, thus serving active TGFβ for metabolic usage. However, since TGFβ activators TSP-1, as well as MMP-9 and MMP-2, could be identified especially in medium αMEM+8%PL; also, reasons other than inactivation may have accounted for altered TGFβ metabolism. Interestingly, Hahn et al. found increased glycolytic activity of adipose-tissue-derived MSCs after TGFβ exposure [66]. This supports the aforementioned assumption of increased energy production by glycolysis for cells grown in media with high content of αMEM+8%PL, which had a significantly higher TGFβ concentration in media. FGF-2 consumption was significantly higher for cells grown in medium 13, but no differences were observed in expression levels of FGF receptors. Since ligand-induced endocytosis is a known negative feedback regulation for several growth factor and hormone receptors [67], downregulation of FGFRs would be more likely than a general lack of FGFR expression. Consumption of PDGF-AA and PDGF-AB/BB was higher for cells cultured in αMEM+8%PL-based media, whereas expression of both isotypes of PDGFRs, PDGFRA and PDGFRB, decreased for cells with rising content of αMEM+8%PL in media. This may be again explained by ligand-induced endocytosis of receptors [67], indicating a lower metabolic usage of PDGF by cells grown in StemMACS^TM^-based media. This hypothesis could be further supported by differences in the binding of PDGF identified during proteomic analyses of cells. Insulin showed important growth-promotion of MSCs in SF media in a study performed by Li et al. [68], while IGF-I and IGF-II have been shown to support osteogenic differentiation of MSCs [69,70]. The IGF system is a well-regulated system including ligands IGF-I, IGF-II, and insulin, receptors IGF1R, IGF2R, and INSR, as well as several IGF-binding proteins. Within this system, crosstalk between ligands and receptors occurs in a way that all ligands can bind to IGF1R and INSR [71], while IGF2R is only bound by IGF-II [72]. In contrast to IGF1R and INSR, which mediate cell growth and survival [71,73], IGF2R accounts for lysosomal degradation of IGF-II, thereby regulating its availability for binding to the other receptors [74]. Expression of IGFR1 and INSR was generally low across cells of all media, assumedly accounted for by receptor-mediated endocytosis. Higher expression was observed for IGF2R, especially for cells grown in media 7 and 10, though expression levels were still low. Decreased IGF2R expression was associated with increased cell proliferation due to enhanced availability of IGF-II [75]. However, during this study, higher proliferation was observed for cells expressing higher IGF2R levels, thus assuming a rather minor role of IGF2R in the regulation of cell proliferation. All in all, given the divergent consumption of several factors and expression of receptors important for cell growth, a combination of both media might have been supportive for MSC proliferation by including factors of both media. This supports the notion that it is not just one or few factors that are solely responsible for MSC proliferation, but a complex interaction with redundancy and possibly synergism between factors exists [34,35].

Aside from receptors for growth factors, the expression of other functionally relevant surface antigens was also analyzed. The diminished therapeutic effect of MSCs is linked to the pulmonary first-pass effect, which traps MSCs in the lung, thereby preventing them from reaching their target sites [76]. Increased homing of MSCs towards bone and reduced trapping of cells in the lung were associated with CD49d expression in a study performed by Kumar et al. [77]. Hence, increased CD49d expression might be of advantage, which was especially observed for cells grown in medium 7. CD49f was described as a stemness marker of MSCs [78] and its expression was linked to higher adipogenic and osteogenic differentiation potential [78,79]. A trend of reduced adipogenic differentiation along with reduced CD49f expression seemed to be also apparent for cells grown with increasing content of StemMACS^TM^, while a diminished osteogenic differentiation could not be clearly ascertained during this study. However, with regard to the differentiation potential of cells, it has to be generally noted that evaluation was only conducted on a visual base. CD146 has been shown to impact MSC potency by affecting amongst others their immunomodulatory potential [80,81,82,83]. A slightly reduced expression of CD146 was observed for cells grown in StemMACS^TM^-based media 10 and 13, though expression levels were still high. Likewise, a minor reduction in CD9 expression was obtained for cells grown in medium 10, which was associated with decreased pro-angiogenic potential in a study by Kim et al. [84]. Differences in expression of surface antigens were, aside from PDGFRB, most pronounced for CD362, where expression increased for cells grown with increasing content of StemMACS^TM^. CD362 (syndecan-2), expressed by a specific MSC subpopulation, was found to be a suitable marker for MSC isolation, thereby resulting in a more homogenous MSC population [85]. These cells have not only been shown to be equally effective as heterogeneous MSC populations in the treatment of pneumonia [85,86] but CD362 was even identified as a major regulator of therapeutic action regarding treatment of sepsis [87]. CD10 was identified as an upregulated marker during osteogenic differentiation of MSCs as compared to undifferentiated cells [88]. Since expression of CD10 was increased for cells grown in media containing StemMACS^TM^, factors included in this media may have resulted in CD10 upregulation. MSCA1 has been proven to be identical with tissue non-specific alkaline phosphatase (ALP) [89]. In a study performed with MSCs from periosteal tissue, MSCA1-positive populations have been linked to higher osteogenic differentiation potential due to increased expression of osteogenic markers ALP and RUNX2 [90]. MSCA1 expression levels during this study were especially high for cells cultured in medium 7 and accordingly low for those of medium 4, though expression was generally highly dependent on MSC donors and showed strong variation among different donors.

MSC secretome includes several factors that are known to affect various cellular programs associated with MSC potency. While some factors differed gradually between the growth conditions, others showed a clear pattern associated with the respective growth condition. Leptin, PDGF-AA and sCD40L were found only in SF/XF cultures. In contrast, FGF-2, TGFβ and insulin were only secreted by PL-expanded MSCs. For some factors, e.g., OPG, OC, OPN, or IL-6, which are relevant for bone formation, the highest concentrations in the supernatant of MSCs were reached in cultures combining both supplements. Given that these factors are important mediators, the altered secretome might be associated with different functionality. The potential implications of a change in various factors are discussed below.

With regard to immunomodulatory actions of MSCs, TGFβ affects not only the migration of macrophages/monocytes in a concentration-dependent manner [91,92] but also directed macrophage polarization towards an anti-inflammatory M2-like phenotype [93]. Furthermore, atopic dermatitis could be mitigated by TGFβ-dependent suppression of TNFα secretion from mast cells [94]. TSG-6-dependent modulation of macrophages was described by several groups, leading to protection of renal tubular cells [95], accelerated wound healing and reduced fibrosis [96] as well as alleviated burn-induced inflammation [97] and zymosan-induced peritonitis [98]. Macrophage polarization was also shown to be affected by the chemokine MCP-1. Polarization of anti-inflammatory IL-10^+^ macrophages has been shown in a colitis model [99], and neuroprotective effects in spinal cord injury, as well as improved wound healing, were associated with MCP-1-dependent recruitment of macrophages and their polarization towards a reparative phenotype [100,101]. Anti-inflammatory effects were additionally linked to MSC-secreted HGF in the treatment of radiation-induced injuries, psoriasis and bronchiolitis obliterans [102,103,104,105]. MSC-conditioned media containing high levels of IL-6 resulted in improved wound healing [106] which was associated with the conversion of macrophages from pro-inflammatory M1 to the anti-inflammatory M2 phenotype by Liu et al. [107]. Furthermore, IL-6 was linked to the suppression of T cell proliferation [108] and prevention of neutrophil apoptosis [109], but also to autoimmune diseases in the case of disturbed IL-6 balance [110]. Modulation of neutrophil migration was reported for MSC-derived IL-6, IL-8, GM-CSF and macrophage-inhibitory factor [111]. GROα, secreted by MSCs, was identified as a key factor in preventing GvHD, namely by enrichment of myeloid-derived suppressor cells (MDSC) [112]. sCD40L was described with regard to immunosuppression in cancer patients where elevated sCD40L levels were associated with increased numbers of MDSCs [113]. During this study, the secretion of immunomodulatory factors varied significantly between cells grown in different media. While cells of all media may affect the polarization of macrophages by different factors (e.g., TGFβ for cells grown in medium 1, MCP-1 and IL-6 for cells grown in medium 4, TSG-6 for cells grown in media 7, 10, and 13), modulation of neutrophils may be especially increased for cells cultured in medium 7, secreting high levels of IL-6 and IL-8. Especially cells grown with high percentages of StemMACS^TM^ showed enhanced GROα as well as sCD40L secretion and thus may have increased potential in the treatment of GvHD. Markedly increased levels of secreted HGF were obtained for cells cultured in media 7 and 10, indicating beneficial effects in different inflammatory diseases. Notably, most of the aforementioned studies used gene modification for HGF overexpression, which may be also achieved by expansion in distinct growth media, as shown by the results of this study. Suga et al. delineated the upregulation of HGF secretion by FGF-2 [114]. FGF-2 was also identified in media during this study and increased with the content of StemMACS^TM^. Hence, FGF-2 in media may have influenced HGF secretion, though also other factors must have played a role since no upregulation of HGF was observed for cells grown in 100% StemMACS^TM^—the media containing the highest concentrations of FGF-2.

Proper bone regeneration requires a balanced homeostasis between bone formation and bone resorption. This process is mainly regulated by the activity of two cell types: osteoblasts, responsible for bone formation, and bone-resorbing osteoclasts. Interaction between these cells occurs amongst others by receptor activator of nuclear factor κ B (RANK), expressed by osteoclast precursors and osteoclasts, and RANK ligand (RANKL), expressed by osteoblasts. Binding of RANKL by its receptor RANK leads to the maturation of osteoclasts and activation of bone resorption [115]. OPG, secreted by osteoblasts [116], can prevent osteoclast activation by acting as a decoy receptor for RANKL, thereby inhibiting catabolic events [117]. OPG secretion by MSCs was reported by Park et al. [118] and others and showed promising results in the treatment of disorders relying on disturbed bone metabolism like osteosarcoma [119], but also in inflammatory diseases like rheumatoid [120] or psoriatic arthritis [121], respectively. Disturbed bone formation has been associated with global inhibition of IL-6 during different phases of fracture healing in a study by Prystaz et al. [122]. The factor PDGF-AA promotes osteogenesis by inducing osteoblast differentiation of precursors like MSCs [123], a process that has been shown to be enhanced in MSCs overexpressing leptin [124,125]. Moreover, OC, OPN, RANTES and also MMP-1 were found to be important during osteogenic differentiation of MSCs [126,127,128,129], and Nakamura et al. proposed OC as a predictive marker for this process [130]. In contrast to the aforementioned factors, DKK1 prevents generation of osteoblasts by inhibiting Wnt signaling and thus impedes maturation of osteoblasts from precursors like MSCs [131]. Although TGFβ-mediated induction of MSC migration to bone resorptive sites has been identified as a major regulator during bone remodeling [132,133], differentiation of osteoblasts was inhibited by this factor [134]. During this study, secretion of factors like leptin, PDGF-AA and RANTES was generally low and only obtained for cells grown in media with high percentages of StemMACS^TM^, whereas expression of MMP-1 decreased for those cells. High levels of OC, IL-6, and OPG were observed for cells grown in media 4 and 7. While expression of CD10 and MSCA1, both associated with osteogenic differentiation of MSCs [88,90], was highest for cells grown in medium 7, those of medium 4 showed reduced expression levels and additionally a significantly higher secretion of DKK1, a factor impeding with osteogenic differentiation. These results generally propose high support of osteogenic differentiation by MSCs grown in media containing both αMEM+8%PL and StemMACS^TM^, which, however, need to contain equal parts of each media. Interestingly, OPN secretion was generally low for cells grown in media with high percentages of αMEM+8%PL (media 1+4), which seemed to have higher in vitro adipogenic differentiation capacity. In accordance with these results, Chen et al. described increased adipogenic differentiation by genetic ablation of OPN [128].

Angiogenesis is a major part of regenerative processes and is responsible for the supply of oxygen and nutrients to newly formed tissue. Support of angiogenesis has been linked to many MSC-derived factors. FGF-2 was associated with improved angiogenesis by supporting the proliferation and tube formation of endothelial cells [135,136] and enhanced expression of pro-angiogenic factors by MSCs [137]. However, the expression of VEGF by endothelial cells was also found to be induced by FGF-2 [138]. VEGF promoted differentiation of endothelial cells from progenitors [139], and increased expression levels were associated with therapeutic efficacy in a myocardial infarction model [140]. Interestingly, a combined application of FGF-2 and VEGF increased tube formation of endothelial cells as compared to both factors alone [135], and a combination of MSC-derived VEGF, MCP-1, and IL-6 was identified as a driving mediator of angiogenesis in a hindlimb ischemia model [141]. Although HGF had no major effect on angiogenesis in the latter study performed by Kwon et al. [141], several studies described HGF-dependent amelioration of angiogenesis and blood vessel restoration [142,143,144,145,146]. Furthermore, pro-angiogenic properties have been shown for follistatin [147], leptin [148], and HB-EGF [149]. In contrast to all pro-angiogenic factors mentioned so far, TSP-1 possesses anti-angiogenic properties [150]. MSC treatment has also shown promising results in many neurodegenerative disorders where neuroprotection and neurological regeneration are of major relevance. MSC-derived FGF-2 supported neurogenesis by inducing proliferation of neural progenitor cells [136], and HGF showed neuroprotection and improved neuronal recovery [142,151,152,153]. Furthermore, TSP-1 was associated with neuroprotection and promotion of neurite outgrowth [154,155] which was also described for MSC-derived fractalkine [156]. During this study, high secretion of pro-angiogenic factors like follistatin, IL-6, HGF, and MCP-1 was observed for cells grown in media mixtures of αMEM+8%PL and StemMACS^TM^, while secretion of VEGF-A seemed to be only reduced for cells lacking any αMEM+8%PL (medium 13). Notably, only cells grown in medium 1 secreted FGF-2 at all, though secretion levels were generally low—a phenomenon also observed by Aizman et al., who described low secretion but high intracellular depots of FGF-2 [136]. In contrast, a trend of decreasing secretion with increasing content of StemMACS^TM^ in media was obtained for TSP-1. Therefore, support of angiogenesis may be improved for cells grown in media 10, showing high secretion of follistatin, VEGF-A, HGF and MCP-1 and reduced secretion of anti-angiogenic factor TSP-1. With regard to neurological regeneration, cells cultured in medium 7, secreting high levels of HGF and TSP-1, may be of advantage.

Wound healing not only demands re-vascularization of tissue but also its re-epithelialization. Amongst other cell types, keratinocytes contribute to this process by proliferation and migration [157], and HB-EGF has been shown to improve keratinocyte migration [158]. However, regenerative mechanisms during wound healing can be disturbed, thereby leading to fibrosis, a process that is characterized by excessive scar formation. HGF and TSG-6 have been shown to prevent fibrosis [96,105,114,142,159,160,161,162], whereas TGFβ was identified as a key factor in mediating scar formation, thereby leading to several pathogenic disease patterns [163]. Since secretion of TGFβ was only observed for cells grown in αMEM+8%PL (medium 1) and only 5% of media StemMACS^TM^ (medium 4) completely abolished this secretion, factors derived from StemMACS^TM^ must have switched TGFβ metabolism, as already discussed above. This may direct MSCs towards an anti-fibrotic phenotype, which can be further substantiated by the high secretion of anti-fibrotic factors TSG-6 and HGF by these cells.

Secretion of insulin was only observed for cells grown in medium 1 and differentiation of MSCs towards insulin-producing cells has been shown by expansion under specific growth conditions, bearing potential for treatment of diabetes [164,165]. However, all in all, it has to be noted that although the secretion of several factors could be shown during this study, technical artifacts of the assay cannot be excluded.

The migratory capacity of MSCs allows for homing toward sites of injury, which is a crucial feature during many therapeutic applications. MMPs are major regulators of cell migration that do not only act by turnover of extracellular matrix (ECM) proteins but also by affecting growth factors, cytokines, chemokines, and surface proteins [65,166,167,168,169,170]. Expression of MMP-1 and MMP-2 has been shown to be essential for the migration of MSCs [129,171,172,173,174]. MCP-3 and HB-EGF were identified as chemotactic factors [175,176], and secretion of MCP-1 by MSCs was described with regard to their enhanced migration [177]. Moreover, TGFβ has been shown to induce MSC migration [133], and this factor was associated with the induction of MCP-1 expression in different cell types [178,179,180]. The migration of cells grown in medium 4 was significantly increased during this study. High expression of MMP-1 and MMP-2 was observed for these cells in addition to high secretion levels of MCP-3 and HB-EGF, which may have augmented migration in an autocrine manner by acting as chemoattractants. In line with the aforementioned studies connecting TGFβ with MCP-1, cells grown in medium 4 consumed significantly more TGFβ which may have induced MCP-1 expression, thus enhancing MSC migration. Notably, the migratory potential of cells grown with high percentages of StemMACS^TM^ (media 10+13) was considerably impaired as these cells were not able to fully close the gap during scratch wound migration assay. Becker et al. linked the decreased migratory potential of MSCs to the high confluence of the cell cultures at the time point of harvesting [173]. During this study, high harvesting densities were observed for cells grown in media 7, 10, and 13. In addition, increasing the content of StemMACS^TM^ in media led to strong adhesion properties of respective cells, not only towards the surface of the culture vessel but also between adjacent cells, resulting in big cell clumps. Hence, cells grown in media 10 and 13 may have been impaired in their migration by their adhesion properties.

The results of this study indicated an effect of growth media on the functional characteristics of MSCs such as their differentiation potential or migratory capacity. Furthermore, differential secretion of several factors associated with regenerative and immunomodulatory properties could be identified depending on culture conditions, thus potentially leading to divergent functionality. However, the actual potency of MSCs needs to be further elucidated in specific disease conditions where distinct factors and cell characteristics might be of particular relevance. However, small extracellular vesicles (sEVs) secreted by MSCs have shown equal therapeutic efficacy as MSCs [181,182] while bearing several advantages as compared to their parental cells (e.g., their potential to cross biological barriers) [183]. Since the effective isolation of sEVs from MSCs have been recently shown by our group [184], the characteristics and potency of sEVs derived from MSCs cultured in different media may be addressed in the future.

## 5. Conclusions

Overall, our study demonstrates that in addition to the biological pleiotropy of MSCs, even seemingly small changes in the expansion conditions, e.g., modification of supplements in the growth media, can result in substantial changes in cell characteristics. We cannot conclude whether one approach (XF/PL-based expansion alone, SF/XF medium alone, or a combination thereof) is generally superior. Rather, our experiments reveal that the choice of the growth-promoting supplement can result in alterations in MSC phenotype and function. The choice might depend on the targeted properties and the intended use of the clinical MSCs. While specific MSC properties obtained during varying culture conditions may be favorable for different applications, the stronger proliferation of cells grown in combined media might help in reducing manufacturing costs due to a lower expansion time required to obtain clinically relevant cell numbers. Nevertheless, for practical reasons, the regulatory burden must not be disregarded when using combined media. Hence, the results should underline that the type and concentration of the MSC-growth-promoting supplement are critical components of the GMP manufacturing process of MSCs, which requires careful validation in case of change.

## Figures and Tables

**Table 1:** Composition of expansion media 1 to 13 for MSC cell cultures. MSCs were expanded in different ratios (1–13) of the media αMEM supplemented with 8% platelet lysate (αMEM+8%PL) and StemMACS^TM^ MSC Expansion Media Kit XF (StemMACS^TM^). Media used for isolation and expansion of MSCs were highlighted in bold and color (medium 1 in black, medium 4 in blue, medium 7 in green, medium 10 in red, and medium 13 in violet).

	1	2	3	4	5	6	7	8	9	10	11	12	13
**aMEM+8%PL** **[%]**	**100**	**99**	**97.5**	**95**	**90**	**75**	**50**	**25**	**10**	**5**	**2.5**	**1**	**0**
**StemMACS^TM^** **[%]**	**0**	**1**	**2.5**	**5**	**10**	**25**	**50**	**75**	**90**	**95**	**97.5**	**99**	**100**

## Data Availability

The raw data supporting the conclusions of this article will be made available by the authors.

## Supplementary Materials

The following supporting information can be downloaded at: https://www.mdpi.com/article/10.3390/cells12162105/s1, Figure S1: Gene ontology (GO) terms of pathway and process enrichment analyses for MSC grown in media 1 and 13; Figure S2: Concentration of different factors in growth media 1, 4, 7, 10, and 13; Table S1: Staining details of standard panel for flow cytometric analysis of cells grown in media 1 to 13 using FACScan^TM^ system; Table S2: Staining details of standard panel for flow cytometric analysis of cells grown in media 1, 4, 7, 10, and 13 using FACSCelesta^TM^ Cell Analyzer; Table S3: Staining details of extended panel for flow cytometric analysis of cells grown in media 1, 4, 7, 10, and 13 using FACSCelesta^TM^ Cell Analyzer; Table S4: Proteomic analysis of cells; Table S5: Proteomic analysis of media.
