# Supplementary material for: Effect of Expansion Media on Functional Characteristics of Bone Marrow-Derived Mesenchymal Stromal Cells
Source: Cells. 2023 Aug 19;12(16):2105. doi: 10.3390/cells12162105 (PMC10453497; doi:10.3390/cells12162105)
Supplement: Supplementary file 1 [file cells-12-02105-s001.zip › cells-2540249 Supplementary Material.pdf]

***Table S1: Staining details of standard panel for flow cytometric analysis of cells grown in media 1 to 13 using FACScan™ system.*** *Cells were stained with fluorescein isothiocyanate (FITC) -, phycoerythrin (PE) - or peridinin chlorophyll (PerCP) –coupled antibodies in staining approaches 1-5. An isotype control (IgG) was included for each fluorochrome.*

| **Number** | **FITC** | **PE** | **PerCP** |
|---|---|---|---|
| **1** | IgG | IgG | IgG |
| **2** | CD90 | CD34 | CD14 |
| **3** | CD73 | | CD45 |
| **4** | CD105 | | |
| **5** | MHC II | | |

***Table S2: Staining details of standard panel for flow cytometric analysis of cells grown in media 1, 4, 7, 10, and 13 using FACSCelesta™ Cell Analyzer.*** *Cells were stained with brilliant violet (BV) 421-, BV786-, fluorescein isothiocyanate (FITC) – or phycoerythrin (PE) -coupled antibodies in staining approaches 1-5. Different isotype controls (IgG) were included for each fluorochrome.*

| **Number** | **BV421** | **BV786** | **FITC** | **PE** |
|---|---|---|---|---|
| **1** | IgG | IgG | IgG | IgG |
| **2** | CD14 | | MHC II | MHC I |
| **3** | | CD45 | CD90 | CD34 |
| **4** | IgG | | IgG | |
| **5** | CD105 | | CD73 | |

***Table S3: Staining details of extended panel for flow cytometric analysis of cells grown in media 1, 4, 7, 10, and 13 using FACSCelesta™ Cell Analyzer.*** *Cells were stained with brilliant violet (BV) 421-, BV786-, fluorescein isothiocyanate (FITC) -, alexa fluor (AF) 488-, phycoerythrin (PE) -, peridinin chlorophyll (PerCP) -, allophycocyanin (APC) -, AF647- or APC-R700–coupled antibodies in staining approaches 1-14. Different isotype controls (IgG) were included for each fluorochrome.*

| **Number** | **BV421** | **BV786** | **FITC/AF488** | **PE** | **PerCP** | **APC/AF647** | **APC-R700** |
|---|---|---|---|---|---|---|---|
| **1** | IgG | IgG | | IgG | | IgG | IgG |
| **2** | | CD44 | | MHC I | | | CD13 |
| **3** | CD146 | | | | | CD140a | |
| **4** | IgG | IgG | IgG | IgG | IgG | IgG | |
| **5** | CD220 | CD140b | GLUT4 | CD221 | CD3 | STRA6 | |
| **6** | IgG | IgG | IgG | IgG | | IgG | |
| **7** | CD81 | CD63 | CD362 | CD9 | | MSCA1 | |
| **8** | IgG | IgG | IgG | IgG | | IgG | |
| **9** | CD49f | CD10 | GLUT3 | CD51 | | GLUT1 | |
| **10** | IgG | IgG | IgG | IgG | IgG | IgG | |
| **11** | CD49e | | CD29 | CD49a | CD36 | CD49d | |
| **12** | CD49c | CD61 | CD271 | | | CD31 | |
| **13** | IgG | | IgG | IgG | IgG | IgG | |
| **14** | EGFR | | CD222 | CD333 | CD331 | CD332 | |

**A**

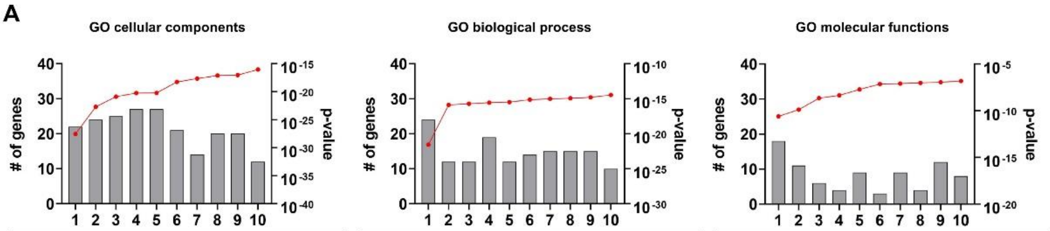


| | | |
|---|---|---|
| 1 | GO:0072562 | blood microparticle |
| 2 | GO:0005788 | endoplasmic reticulum lumen |
| 3 | GO:0062023 | collagen-containing extracellular matrix |
| 4 | GO:0031012 | extracellular matrix |
| 5 | GO:0030312 | external encapsulating structure |
| 6 | GO:0031983 | vesicle lumen |
| 7 | GO:0031091 | platelet alpha granule |
| 8 | GO:0034774 | secretory granule lumen |
| 9 | GO:0060205 | cytoplasmic vesicle lumen |
| 10 | GO:0031093 | platelet alpha granule lumen |

| | | |
|---|---|---|
| 1 | GO:0050878 | regulation of body fluid levels |
| 2 | GO:0030193 | regulation of blood coagulation |
| 3 | GO:1900046 | regulation of hemostasis |
| 4 | GO:0042060 | wound healing |
| 5 | GO:0050818 | regulation of coagulation |
| 6 | GO:0061041 | regulation of wound healing |
| 7 | GO:0007596 | blood coagulation |
| 8 | GO:0050817 | coagulation |
| 9 | GO:0007599 | hemostasis |
| 10 | GO:0030195 | negative regulation of blood coagulation |

| | | |
|---|---|---|
| 1 | GO:0050839 | cell adhesion molecule binding |
| 2 | GO:0005201 | extracellular matrix structural constituent |
| 3 | GO:0070325 | lipoprotein particle receptor binding |
| 4 | GO:0060228 | phosphatidylcholine-sterol O-acyltransferase activator activity |
| 5 | GO:0005178 | integrin binding |
| 6 | GO:0070653 | high-density lipoprotein particle receptor binding |
| 7 | GO:0061135 | endopeptidase regulator activity |
| 8 | GO:0048407 | platelet-derived growth factor binding |
| 9 | GO:0004857 | enzyme inhibitor activity |
| 10 | GO:0002020 | protease binding |

**B**

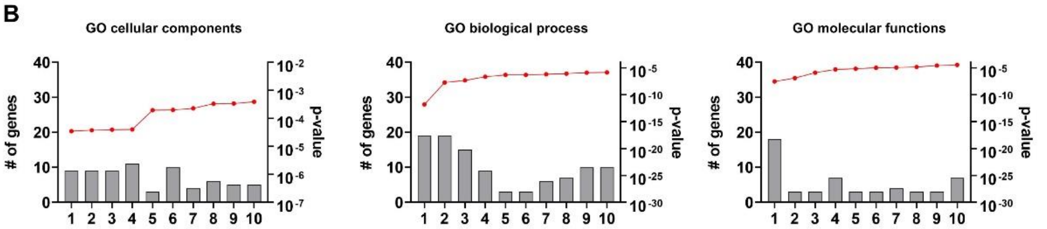


| | | |
|---|---|---|
| 1 | GO:0034774 | secretory granule lumen |
| 2 | GO:0060205 | cytoplasmic vesicle lumen |
| 3 | GO:0031983 | vesicle lumen |
| 4 | GO:0015629 | actin cytoskeleton |
| 5 | GO:0071682 | endocytic vesicle lumen |
| 6 | GO:0005635 | nuclear envelope |
| 7 | GO:0000502 | proteasome complex |
| 8 | GO:0101002 | ficolin-1-rich granule |
| 9 | GO:1905368 | peptidase complex |
| 10 | GO:1904813 | ficolin-1-rich granule lumen |

| | | |
|---|---|---|
| 1 | GO:0044283 | small molecule biosynthetic process |
| 2 | GO:0043603 | amide metabolic process |
| 3 | GO:0032787 | monocarboxylic acid metabolic process |
| 4 | GO:1901617 | organic hydroxy compound biosynthetic process |
| 5 | GO:0009753 | response to jasmonic acid |
| 6 | GO:0071395 | cellular response to jasmonic acid stimulus |
| 7 | GO:0006081 | cellular aldehyde metabolic process |
| 8 | GO:0033559 | unsaturated fatty acid metabolic process |
| 9 | GO:0046394 | carboxylic acid biosynthetic process |
| 10 | GO:0016053 | organic acid biosynthetic process |

| | | |
|---|---|---|
| 1 | GO:0016491 | oxidoreductase activity |
| 2 | GO:0018636 | phenanthrene 9,10-monooxygenase activity |
| 3 | GO:0047086 | ketosteroid monooxygenase activity |
| 4 | GO:0051087 | protein-folding chaperone binding |
| 5 | GO:0047023 | androsterone dehydrogenase activity |
| 6 | GO:0047044 | androstan-3-alpha,17-beta-diol dehydrogenase activity |
| 7 | GO:0016628 | oxidoreductase activity, acting on the CH-CH group of donors, NAD or NADP as acceptor |
| 8 | GO:0032052 | bile acid binding |
| 9 | GO:0004032 | alditol:NADP+ 1-oxidoreductase activity |
| 10 | GO:0016705 | oxidoreductase activity, acting on paired donors, with incorporation or reduction of molecular oxygen |

observed gene count

p-value

***Figure S1: Gene ontology (GO) terms of pathway and process enrichment analyses for MSC grown in media 1, and 13.*** *MSC, isolated and expanded in αMEM+8%PL (medium 1; black) or StemMACS™ (medium 13; violet), were used for proteomic analyses. Differentially expressed proteins between cells grown in media 1* ***(A)*** *and 13* ***(B)*** *were analyzed in a pathway and process enrichment analysis. The proteins were associated with divergent cellular components, biological processes and molecular functions, where different numbers of genes (observed gene count; gray bars) were identified for each GO term with a specific false discovery rate (p-value; red dotted line).*

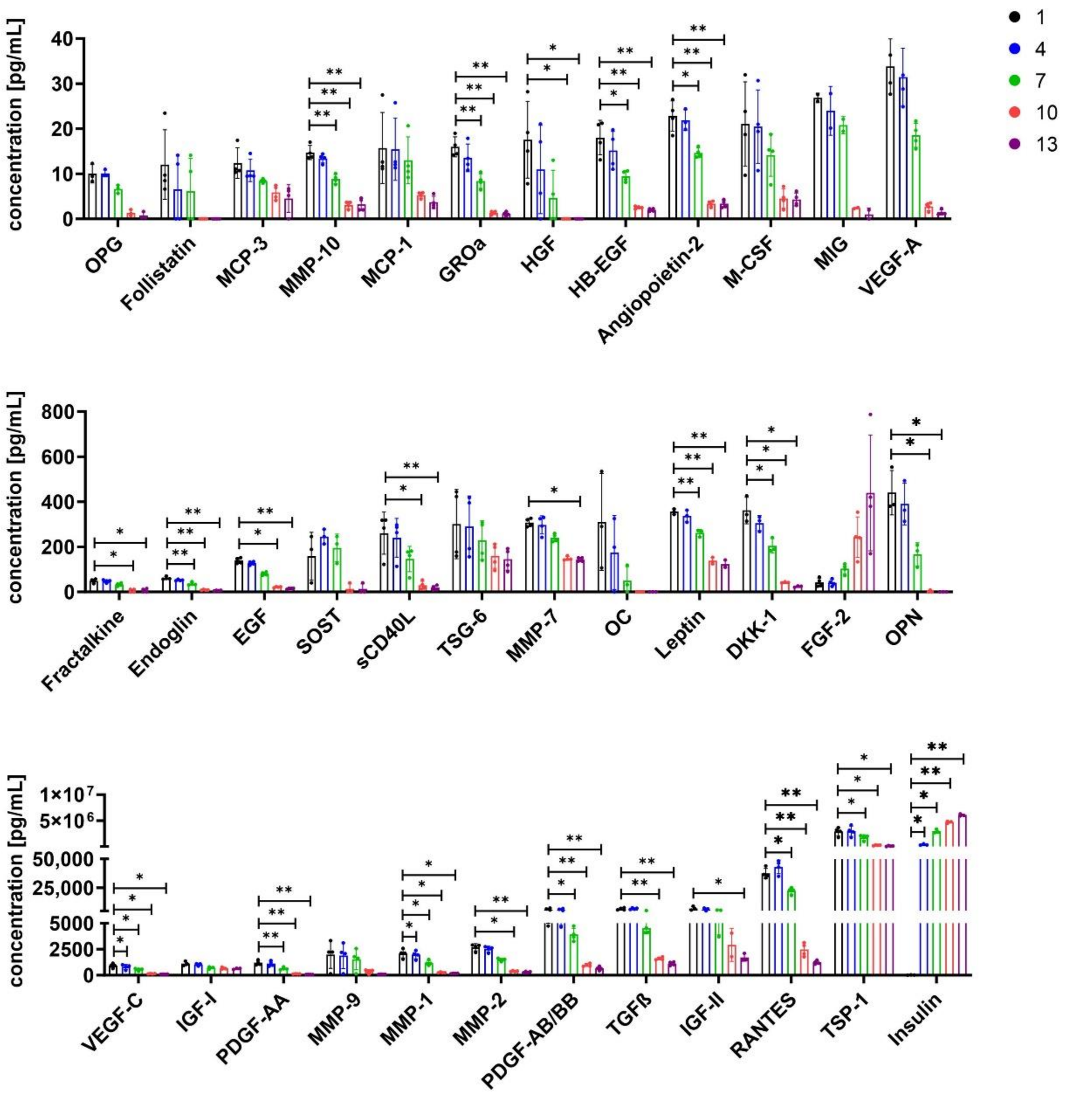


*Figure S2:* ***Concentration of different factors in growth media 1, 4, 7, 10, and 13.*** *The concentration of several factors was analyzed in media 1 (black), 4 (blue), 7 (green), 10 (red), and 13 (violet).*
