# Supplementary figures and images for: Effect of Expansion Media on Functional Characteristics of Bone Marrow-Derived Mesenchymal Stromal Cells
Source: Cells. 2023 Aug 19;12(16):2105. doi: 10.3390/cells12162105 (PMC10453497; doi:10.3390/cells12162105)

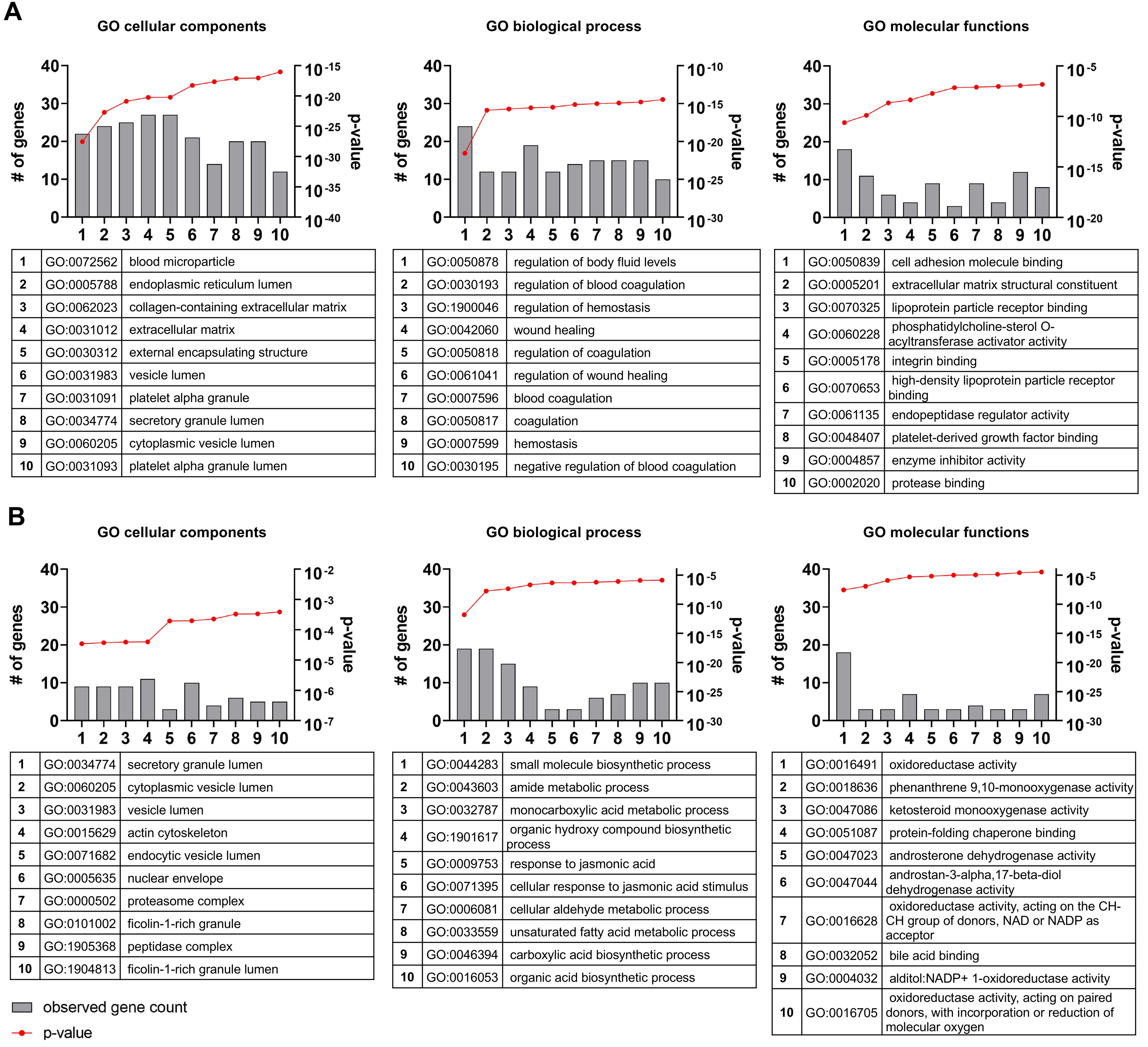

Supplement: Supplementary file 1 [file cells-12-02105-s001.zip › cells-2540249 Figure S1.jpg]

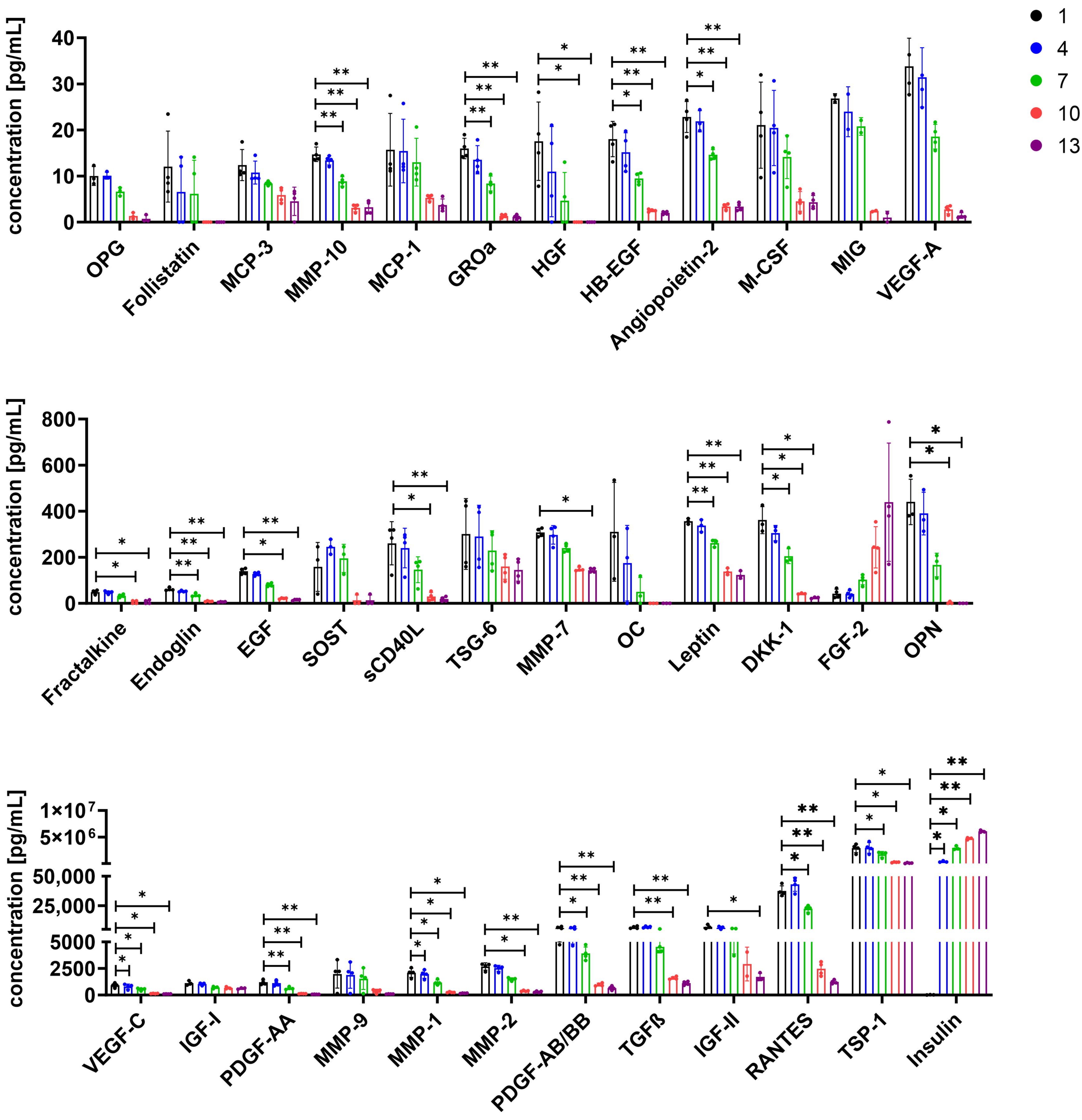

Supplement: Supplementary file 1 [file cells-12-02105-s001.zip › cells-2540249 Figure S2.jpg]
